# T Cell-Mediated Beta Cell Destruction: Autoimmunity and Alloimmunity in the Context of Type 1 Diabetes

**DOI:** 10.3389/fendo.2017.00343

**Published:** 2017-12-05

**Authors:** Adam L. Burrack, Tijana Martinov, Brian T. Fife

**Affiliations:** ^1^Department of Medicine, Center for Immunology, University of Minnesota Medical School, Minneapolis, MN, United States

**Keywords:** type 1 diabetes, immunology, autoimmune diseases, transplantation immunology, tolerance induction, T cells, alloimmunity

## Abstract

Type 1 diabetes (T1D) results from destruction of pancreatic beta cells by T cells of the immune system. Despite improvements in insulin analogs and continuous blood glucose level monitoring, there is no cure for T1D, and some individuals develop life-threatening complications. Pancreas and islet transplantation have been attractive therapeutic approaches; however, transplants containing insulin-producing cells are vulnerable to both recurrent autoimmunity and conventional allograft rejection. Current immune suppression treatments subdue the immune system, but not without complications. Ideally a successful approach would target only the destructive immune cells and leave the remaining immune system intact to fight foreign pathogens. This review discusses the autoimmune diabetes disease process, diabetic complications that warrant a transplant, and alloimmunity. First, we describe the current understanding of autoimmune destruction of beta cells including the roles of CD4 and CD8 T cells and several possibilities for antigen-specific tolerance induction. Second, we outline diabetic complications necessitating beta cell replacement. Third, we discuss transplant recognition, potential sources for beta cell replacement, and tolerance-promoting therapies under development. We hypothesize that a better understanding of autoreactive T cell targets during disease pathogenesis and alloimmunity following transplant destruction could enhance attempts to re-establish tolerance to beta cells.

## Introduction

Pancreatic beta cells are destroyed by T cells of the immune system, precipitating type 1 diabetes (T1D). Unfortunately, preventing beta cell destruction in at-risk individuals has proven challenging. Despite a working knowledge of genetic risk factors associated with T1D (1), determining specific beta cell targets and preventing beta cell destruction by autoreactive T cells remains elusive. To develop a successful approach to protect beta cells, we must understand how and why T cells are directed to specifically destroy insulin-producing cells in the pancreas while sparing adjacent hormone-producing cells including alpha, delta, and epsilon cells. There may be at least two paths to protect beta cells from T cell-mediated death. The first approach is to control or regulate effector T cell responses, and the second is to enhance beta cell survival or resistance to T cell-mediated death.

The first section of this review outlines our current understanding of the pathogenesis of autoimmune diabetes. We describe the process by which insulin-producing beta cells are destroyed and contrast the roles of CD4^+^ and CD8 T cells during autoimmune pathogenesis. We compare T1D pathogenesis in the non-obese diabetic (NOD) mouse to our current understanding of human disease. We also discuss an exciting recent development in the field of autoreactive T cell biology: recognition of neoantigens generated through hybrid peptide fusion or response to neoantigens formed through defective protein translation. Finally, we describe immune tolerance in several forms, including thymic central tolerance, T cell ignorance in the periphery, anergy, and regulatory T cell induction.

The second section of this review briefly describes the necessity for pancreas or islet transplantation to treat severe diabetic complications. With improving glycemic control through insulin injections and continuous glucose monitoring, many T1D individuals live with minimal complications (2, 3). However, some T1D individuals develop life-threatening complications including hypoglycemia unawareness and end-stage renal disease. Unawareness of severe hypoglycemia is a primary indicator for pancreas or islet transplantation and is often combined with kidney transplantation to treat renal failure.

The third section of this review focuses on islet replacement strategies and briefly outlines beta cell regeneration. The two primary avenues for beta cell replacement are transplantation of cadaveric islets or induced pluripotent stem cell (iPS)-derived beta cells. While there has been considerable progress in both strategies, a cure for established T1D must also involve targeted immunotherapy. This approach must inhibit memory autoreactive T cells and naive allograft-reactive immune responses. In the third section of this review, we describe allorecognition, or how T cells “see” transplants, focusing on pancreatic islet transplantation. We describe two categories of allorecognition by T cells in transplant recipients: direct recognition of donor major histocompatibility complex (MHC) molecules and indirect recognition of transplant-derived peptides through recipient MHC molecules. We also discuss the challenges of transplant tolerance in the NOD mouse and human T1D islet allograft recipients. Recent evidence suggests that the presence of autoimmunity acts as an “adjuvant,” accelerating and strengthening the conventional alloimmune response.

## Autoimmune Diabetes Pathogenesis

Type 1 diabetes is a T cell-mediated autoimmune disease, whereas T2D is the result of peripheral cell resistance to endogenous insulin. The best evidence supporting immune system involvement in T1D are studies reporting lymphocytic infiltrate in the islets of T1D cadaveric donors (4, 5), islet-specific autoantibody production in individuals with T1D (6–8), and identical twin studies in which the twin with T1D rejected islet transplants from their non-diabetic twin (9). Analyses of pancreas sections harvested from individuals with T1D have shown fulminant immune infiltration within individual islets, corroborating a key role for CD4 and CD8 T cells in beta cell destruction (10–12). This is in sharp contrast to pancreas sections from individuals with T2D, who, despite having high levels of systemic inflammatory markers, do not have similar T cell infiltration within pancreatic islets (10–12). Virtually all individuals who develop T1D before the age of 5 years produce insulin-specific autoantibodies (IAAs), suggesting an important role for peptides derived from the insulin molecule in disease pathogenesis (13, 14). Islet autoantibodies are a differential diagnosis marker for T1D versus T2D and arise from autoreactive B cell and autoreactive CD4 T cell interactions. Human leukocyte antigens (HLAs) class II alleles DR4, DQ8, and DQ2 confer the highest genetic risk for T1D in human patients (15). This strong HLA II allele association with T1D suggests that HLA II-restricted CD4 T cells play a key role in disease pathogenesis. CD4 T cells can provide “help” to B cells and stimulate antibody production as noted above, as well as promote responses by effector CD8 T cells, and stimulate islet-resident macrophages (16, 17). With this in mind, autoreactive CD4 T cells represent an active area of research and clinical interest for therapies. Developing antigen-specific tolerance-promoting methods to inhibit autoreactive CD4 T cells is the focus of the first section of this review.

### The NOD Mouse Model of T1D

The NOD mouse was first characterized at the Shionogi Research Laboratories in Aburahi, Japan, by Makino et al. (18). The NOD mouse was developed as a sub-strain of the JcI:ICR mouse strain, which was used to study cataract development (18). The NOD strain exhibited very high fasting blood sugar levels but not cataracts and has been an invaluable tool for T1D research. Depending on the colony, 50–90% of female NOD mice develop spontaneous autoimmune diabetes between 10 and 30 weeks of age (19). Generally, diabetes onset in male NOD mice is much less frequent (20% in the same age range); therefore the majority of studies of autoimmune diabetes utilizing this strain of mice use female diabetic mice (20). This review will focus on spontaneous autoimmune diabetes pathogenesis in NOD mice, although other models of beta cell destruction mediated by T cell receptor (TCR) transgenic T cells targeting ectopically expressed antigen such as in rat insulin promoter (RIP) driving lymphocytic choriomeningitis virus (21) RIP-membrane-bound form of ovalbumin (22) or insulin hemagglutinin (23) have contributed extensively to our understanding of T1D and are discussed elsewhere (24). Studies in the NOD mouse demonstrate a strong dependence on MHC class II allele I-A^g7^ and the requirement of CD4 T cells (25), CD8 T cells (26), and B cells (27, 28) for autoimmune diabetes. Interestingly, diabetes-associated MHC II, I-A^g7^ does not precipitate diabetes when expressed in non-autoimmune-prone B6 mice (29), but NOD mice engineered to express MHC class II alleles other than I-A^g7^ are protected from disease development (30). Collectively, these findings suggest that I-A^g7^ is necessary, but not sufficient, for autoimmune diabetes. The roles of CD4 T cells, CD8 T cells, and B cells in diabetes pathogenesis are discussed below.

**Figure 1:**
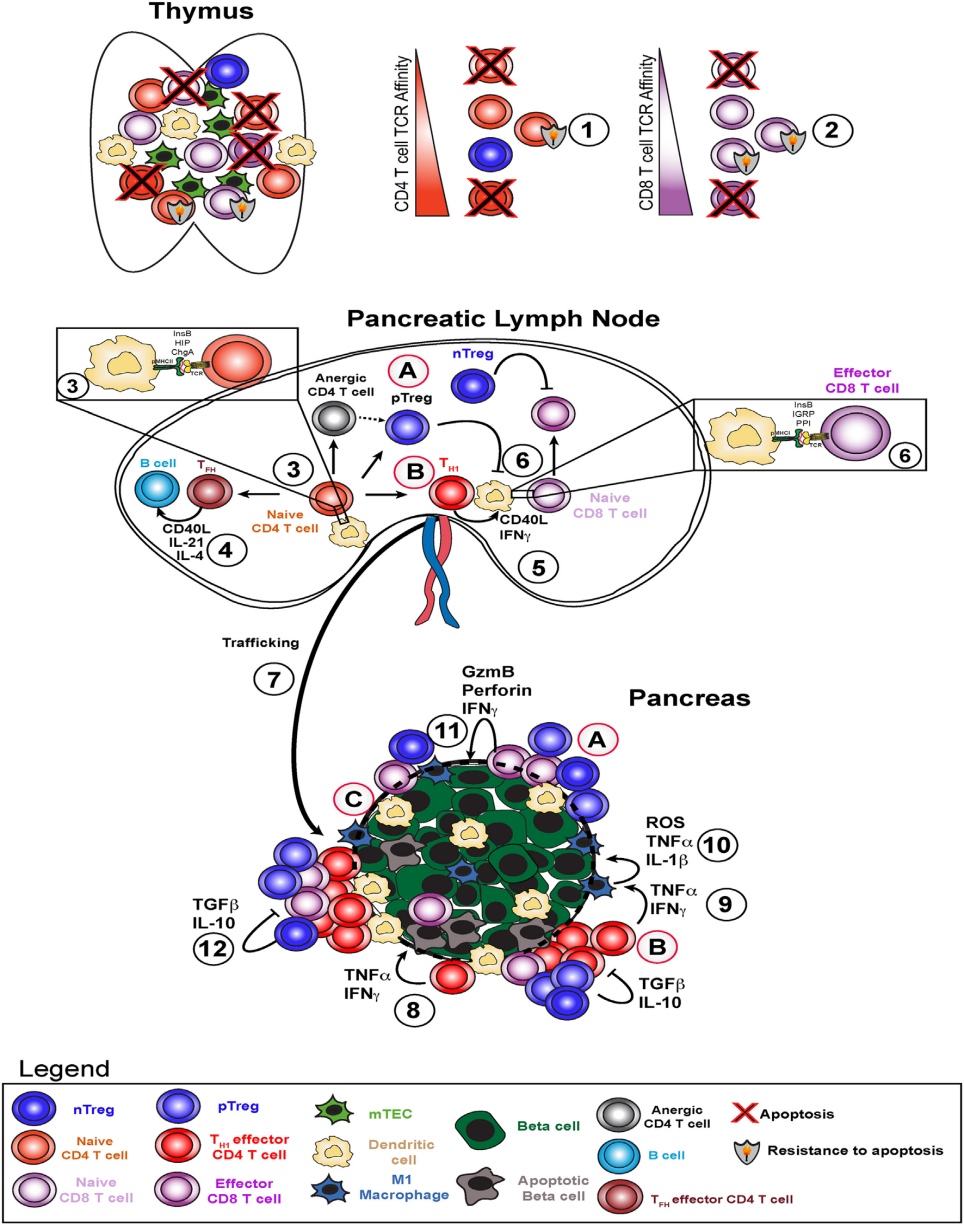
Type 1 diabetes pathogenesis and potential therapeutic avenues. Type 1 diabetes arises due to failure of several key checkpoints. Defective central tolerance (1 and 2) allows islet-reactive CD4 and CD8 T cells to escape the thymus as naive cells and reach the pancreatic lymph node. In the pancreatic lymph node, autoreactive CD4 T cells interact with dendritic cells presenting islet antigen (3) and can become T helper 1 (T_H1_), T_FH_, pTreg, or anergic cells. T_FH_ cells help B cells produce high affinity islet-specific antibodies (4). T_H1_ cells activate dendritic cells and enhance antigen presentation to islet-specific CD8 T cells (5) to induce effector CD8 T cell skewing (6). T_H1_ cells traffic to the pancreas (7), secrete pro-inflammatory cytokines interferon gamma (IFNγ) and TNFα, and induce beta cell death (8). T_H1_-derived IFNγ and TNFα stimulate M1 macrophages in the islets to produce ROS, TNFα, and IL-1β (9), which in turn amplify beta cell death cycle (10). Resulting inflammation leads to increased CD8 T cell infiltration and direct beta cell killing via perforin and granzyme B (11) and attempts by nTregs and pTregs to dampen this response via TGFβ and IL-10 (12). Potential therapeutic strategies include (A) infusion of *ex vivo* expanded (broadly reactive or pancreas-specific) Tregs, (B) re-educating T_H1_ cells through approaches like peptide-linked apoptotic splenocytes, and (C) promoting beta cell-intrinsic expression of defense molecules *in situ* or engineering transplanted beta cells to be more resistant to T cell-mediated attack.

CD4 T cells are thought to provide help to effector CD8 T cells, stimulate antibody production by B cells, and activate islet-resident M1 macrophages (Figure 1). CD4 T cells are required for diabetes development in NOD mice (31), and either depletion of CD4 T cells (32) or treatment with non-depleting anti-CD4 antibodies prevents diabetes (33). Early research in the NOD mouse model demonstrated that T helper 1 cells transferred to neonatal NOD recipient mice could precipitate diabetes (34). Recent studies in NOD mice and human T1D patients have characterized the diabetogenic CD4 T cells as pro-inflammatory, capable of secreting interferon gamma (IFN-γ) and/or interleukin 17 (35–39). Interestingly, HLA-matched healthy donors may also have CD4 T cells with islet antigen specificity, but in their case, the cell phenotype and functional output is regulatory, with a cytokine profile consisting mainly of IL-10 (35, 36). CD4 T cell targets are peptides restricted to HLA or MHC II and are discussed in further detail below. In human T1D, the available evidence from studies of individual islets from the Network for Pancreatic Organ Donors with Diabetes suggests that beta cell destruction is mediated in large part through direct CD8 T cell contact with beta cells and CD4 T cell-mediated polarization of M1 macrophages (4, 10, 40). CD4 regulatory T cells will be addressed below.

Autoreactive CD8 T cells are activated through interaction with peptides presented by MHC class I and can mediate beta cell death in a contact-dependent manner through perforin and granzyme molecules (Figure 1) (41). MHC class I is required for T1D, with some reports suggesting that CD8 T cell/MHC class I interactions are required only early in disease development (42), whereas others have concluded that MHC class I is required late in diabetes pathogenesis (43). Insulin-specific CD8 T cells are key for diabetes onset in both mouse (44, 45) and humans (46). Even though CD8 T cells are required for disease pathogenesis, due to space limitations, the bulk of this review will focus on the biology of CD4 T cells.

Beta cell death can also be mediated through cytokine production by both CD4 and CD8 T cells within pancreatic islets. Pro-inflammatory cytokines such as TNF-α and IFN-γ are directly toxic to beta cells (Figure 1) (47, 48). These cytokines also activate macrophages to M1 phenotype and stimulate a positive feedback loop, further increasing cytokine production *in situ* and killing more beta cells (Figure 1) (49). In addition, data from mouse and human samples demonstrate that beta cells can express the IFN-γ-inducible chemokine CXCL10, which promotes T cell infiltration and may accelerate beta cell destruction (50, 51). Data from adoptive CD4 T cell transfer model of diabetes in the NOD mouse model suggest that M1 macrophages are required for beta cell destruction in this setting (52). Indeed, it has been demonstrated in the NOD mouse that superoxide production by T cells or macrophages is critical to promote beta cell death and T1D (16) and that loss of superoxide production by macrophages delays diabetes pathogenesis (53). Moreover, transient depletion of islet-infiltrating dendritic cells and macrophages using clodronate-loaded liposomes abrogated T cell infiltration and significantly delayed subsequent diabetes development in liposome-treated mice (54). More recent work has demonstrated a critical role for dendritic cells expressing the Batf3 transcription factor in autoimmune pathogenesis of NOD mice (55). Taken together, these results suggest that antigen presentation to CD4 T cells by dendritic cells and macrophages within pancreatic islets plays a key role in promoting beta cell destruction.

Finally, our current understanding is that B cells act as antigen-presenting cells to both CD4 and CD8 T cells and also produce IAAs (Figure 1) (56). Early studies established that NOD mouse production of IAA peaks between 8 and 12 weeks of age and gradually decreases afterward presumably as beta cell mass decreases (57, 58). In addition, >60% of mice which developed IAA at 3–5 weeks of age develop T1D by week 20, while >50% of IAA-positive mice at 8 weeks of age develop T1D by week 20 (57–59). Translating these results to human patients, as pioneered by Eisenbarth (58), autoantibody responses against multiple different T cell antigens are highly predictive of diabetes onset within 12–36 months in human subjects (1, 8, 60). In addition, recent work from Finland has demonstrated that high proportions of children with IAA and/or multiple autoantibodies against beta cell targets at ages younger than 5 years develop T1D (61). As shown by sibling studies (DAISY, TEDDY), the presence of one known autoantibody response confers a moderate risk level, with risk of imminent development of diabetes increasing exponentially with the detection of each additional autoantibody response.

While analogous experiments have not been performed using human autoreactive T cells and human beta cells in an *in vitro* setting or humanized mouse system, studies in the NOD mouse have elucidated potential mechanisms of beta cell destruction in human T1D, in particular key roles for CD4 and CD8 T cells. However, there are important differences between NOD and human T1D. In particular, there is a gender bias in NOD mice, with higher incidence in female than male mice (19, 20). In contrast, human T1D does not show gender bias, unlike other autoimmune diseases. A full account of the physiology behind this discordance is outside the scope of this review, but may include (a) more synchronous T cell infiltration into pancreatic islets in NOD mice than in at-risk human subjects, (b) the potential for a greater dependence on CD8 T cells in diabetes pathogenesis in human disease (10), and (c) confounding effects of multiple concurrent T cell responses in human patients exposed to the “universe” of viral and bacterial pathogens as opposed to inbred specific pathogen-free NOD mouse colonies.

### Autoimmune Diabetes Antigens and Neoantigens

Diabetes-relevant antigen targets have been defined through the presence of serum autoantibodies, ELISpot assays, proliferation assays, and mouse studies [reviewed in Ref. (62)]. In mice and humans, some of the B cell and T cell antigen targets of T1D are overlapping, but not identical (63). The majority of autoantigens identified in the NOD mouse are peptides from the insulin secretory granules. At the Barbara Davis Center in the late 1980s, Haskins et al. (64, 65) and Wegmann et al. (66) utilized the NOD mouse to generate a series of pancreatic islet secretory granule-specific autoreactive CD4 T cell lines (67). Chief among these, the BDC2.5 CD4 T cell line has been studied extensively (68). Two key transgenic mouse lines were generated including the BDC2.5 TCR transgenic mouse (69) and the islet-specific glucose-6-phosphatase catalytic subunit-related protein (IGRP)-specific CD8.3 transgenic mouse (70). The NOD mouse has proven to be a useful “work horse” model system for studying the pathogenesis and cellular immunology of spontaneous and adoptively transferred T1D. T cell-mediated destruction of beta cells represents an intricate coordination between innate and adaptive lymphocytes, with CD4 T cells occupying a key node in this network, as described above. CD4 T cell epitopes discovered to date include epitopes derived from the insulin B chain (45), chromogranin A (71), and islet amyloid polypeptide (Figure 1) (72, 73). CD8 T cell epitopes include peptides derived from preproinsulin (44, 46), IGRP (70), Zinc transporter 8 (74, 75), and glutamic acid decarboxylase 65 (Figure 1) (76). Of particular importance in both the NOD mouse model system and for translation to the human disease is a peptide derived from amino acids 10–23 of the insulin B chain (InsB10:23). This peptide is required for the development of autoimmune diabetes in the NOD mouse (45). Nakayama et al. determined that a single amino acid substitution in a TCR contact site for both CD4 and CD8 T cells conferred complete protection by altering a dominant immune target within the insulin protein (45). Similarly, we determined that insulin-specific T cell responses were critical in the spontaneous mouse model of diabetes (77). We demonstrated that blocking insulin-specific T cell responses could reverse and even cure diabetes in mice. In addition, re-establishing immune tolerance to proinsulin prevents diabetes onset in NOD mice, but re-establishing tolerance to IGRP_206-214_ does not prevent diabetes in NOD mice (78). Despite these fundamental discoveries, we still do not fully understand antigen hierarchy in T1D patients, likely because multiple different targets may be required for disease in different patients (79).

Exciting recent work from several groups has demonstrated the presence of neoantigens for diabetogenic CD4 T cells. These comprise hybrid peptides or combinations of amino acid sequences derived from two different secretory granule proteins or peptide sequences (80, 81). The frequency of T cell priming events against hybrid peptides during autoimmune pathogenesis is not clear *in vivo*; however, compelling evidence *in vitro* suggests that these cells may play an important role in T1D pathogenesis. It is thought that hybrid peptides are generated exclusively in beta cells and not in the thymus, thus representing “new” targets in the periphery. These targets could be viewed as foreign peptides eliciting a strong immune response. Recent reports also suggest that pancreatic neoantigens could arise from defective ribosomal insulin gene products (DRiPs), which are produced by metabolically stressed beta cells (82). Similarly to hybrid peptides, central tolerance to DRiPs generated by stressed beta cells would be lacking in the thymus. In the presence of inflammation and cell death, T cell responses to such neoantigens would develop in the periphery and could contribute to disease pathogenesis. Table 1 summarizes known autoantigens in T1D development in human subjects and NOD mice and if they are recognized by CD4 or CD8 T cells in the context of the appropriate HLA/MHC molecule.

**Table 1:** Beta cell secretory granule-derived auto antigens.

Protein target	NOD mouse and/or human T1D	CD4 and/or CD8 T cells	Reference
(Pre)proinsulin	Mouse and human	CD4 and CD8	(36, 44, 46, 78)
Insulin	Mouse and human	CD4 and CD8	(44, 45, 76, 83)
Defective ribosomal insulin gene product	Human	CD8	(82)
Insulin hybrid peptides	Mouse and human	CD4	(80, 81)
GAD65	Mouse and human	CD4 and CD8	(84–88)
ZnT8	Mouse and human	CD4 and CD8	(74, 75, 89–92)
Islet antigen-2	Human	CD4 and CD8	(93–95)
Phogrin	Mouse and human	CD4	(96–99)
Islet cell autoantigen 69 kDa	Human	CD4	(100–103)
Chromogranin A	Mouse and human	CD4 and CD8	(71, 104, 105)
Islet amyloid polypeptide	Mouse and human	CD4 and CD8	(72, 73, 106, 107)
Islet-specific glucose-6-phosphatase catalytic subunit-related protein	Mouse and human	CD4 and CD8	(70, 78, 108, 109)

### Mechanisms of Immune Tolerance

There are four broad categories of immune tolerance that could protect beta cells from destruction by autoreactive T cells. First, negative selection during thymic development culls self-reactive T cells during T cell development. Due to this mechanism, autoreactive T cells generally do not survive thymic development. However, diabetes-associated MHC class I and II alleles facilitate the escape of self-reactive lymphocytes from the negative selection process. This escape could be due to several non-mutually exclusive reasons: low thymic expression of islet antigens (110), poor binding of native (non-transcriptionally modified) islet autoantigens to MHC I/II [as suggested in Ref. (111)], and T cell-intrinsic resistance to apoptosis (112) (Figure 1). GWAS studies link allelic variation at the insulin variable number tandem repeat (INS-VNTR) IDDM2 locus with the level of thymic insulin expression and disease development. Protective alleles of the IDDM2 diabetes susceptibility locus promote higher levels of insulin expression in the thymus, which would promote more robust negative selection of insulin-reactive T cells (113). In addition, mice genetically engineered to express lower levels of insulin in the thymus demonstrate correspondingly higher levels of peripheral T and B cell reactivity against insulin (110), and published work indicates that pancreatic lymph nodes of NOD mice contain higher than expected levels of insulin mRNA at 3–5 weeks of age (114). These observations suggest a direct link between the level of extra-pancreatic insulin expression and peripheral lymphocyte reactivity to insulin and point to ineffective negative selection in NOD mice and human patients. Second, immune ignorance occurs if an autoreactive lymphocyte survives thymic development, but does not encounter its cognate antigen in the periphery. The ignorance pathway appears to be an important method for maintenance of B cell tolerance (115). Additional evidence of autoantigen-specific T cell ignorance can be found in the MHC-matched T1D-resistant B6.g7 mouse model (116). A third mechanism of tolerance is a state of antigen-specific unresponsiveness called anergy. CD4 T cell anergy is defined as expression of folate receptor 4 and CD73 and hyporesponsiveness to TCR stimulation (117). While the majority of insulin-specific CD4 T cells in NOD mice are anergic, this form of tolerance is not sufficient o halt diabetes (Figure 1) (116). A fourth mode of immune tolerance relies on thymic-derived and peripheral regulatory CD4 T cells (Tregs) expressing the transcription factor Foxp3 (Figure 1). Foxp3 is the master regulator of Treg fate, stability, and suppressive capacity (118). Mutations in the Foxp3 locus (IPEX in humans and Scurfy in mice) lead to multiorgan autoimmunity and demonstrate a non-redundant role of Foxp3 in maintaining tolerance (118). Recent evidence demonstrates that the augmentation of Treg activity specifically within pancreatic islets may ameliorate diabetes pathogenesis in NOD mice (119). This result suggests that promoting Treg activity specifically within the pancreas may be beneficial in human T1D as well. In addition, Tregs can inhibit effector T cells specific for the same or “linked” peptides. “Linked suppression” refers to the ability of regulatory T cells to suppress activation of effector T cells interacting with the same antigen-presenting cell at the time of Treg-APC interaction. This concept was originally demonstrated by Davies et al. (120) and reviewed in Ref. (121) and has been shown to apply to the murine model of multiple sclerosis, experimental autoimmunity encephalomyelitis, as well (122). In addition, this mechanism has recently been shown to apply to a heart transplant model in mice, in which immune tolerance was induced to multiple distinct foreign MHC molecules (123). As such, we speculate that determination of “linked” peptides to promote CD4 T cell tolerance to islet allografts in autoimmune recipients represents a powerful opportunity to prevent islet allograft rejection in autoimmune recipients.

While several hundred protocols have prevented diabetes in NOD mice, very few of these have successfully reversed disease, and none have yet been translated to standard clinical practice (124, 125). Briefly, tolerance-promoting therapies have generally focused on inhibiting autoreactive T or B cells, decreasing inflammation prior to diabetes onset, or some combination of these approaches. In attempts to restore tolerance in the CD4 T cell compartment, we previously used whole insulin protein coupled to apoptotic cells through the chemical cross-linker ethylene carbodiimide, or ECDI (77). This approach reversed T1D in almost half of the treated mice. ECDI-coupled cells have been used in phase I safety trials for multiple sclerosis and have shown a desirable safety profile (Figure 1) (126). We predict that this approach could be tested for safety and efficacy in T1D. Adoptive transfer of regulatory CD4 T cells can halt diabetes pathogenesis in mice through inhibition of IFN-γ production by islet-infiltrating CD4 and CD8 T cells and decreased islet infiltration by CD8 T cells (127). These findings were translated to the clinic, with encouraging results. Two separate research groups have demonstrated that deficiencies in IL-2 production (128) or the responsiveness of Treg cells to IL-2 (129) may be related to the development of autoimmune diabetes in NOD mice. Two separate groups have adoptively transferred autologous (self-derived) Tregs into new-onset T1D patients to enhance function of endogenous Tregs (Figure 1). A European group isolated and expanded Tregs from T1D patients (130) and then went on to demonstrate preservations of C-peptide in 8 of 12 subjects and reversal of new-onset T1D in 2 patients (131). In addition, a group at UCSF led by Bluestone and colleagues developed a protocol to expand Tregs from T1D patients (132) and then proved safety in phase I clinical trials (133). Several groups have established that Tregs can be isolated, expanded *ex vivo* in the presence of CD3/CD28 stimulation and IL-2, and adoptively transferred into patients (132–134). Transferred Tregs were detectable in blood up to 12 months later, remained phenotypically stable, and had the potential to influence diabetes pathogenesis. Both of these Treg adoptive transfer clinical trials utilized *in vitro* expanded Tregs, not Tregs specific for particular pancreatic target(s). It is not known if targeting particular autoantigens would provide additional protection compared to the current Treg transfer approach. Taken together, these recent clinical trials suggest that adoptive Treg therapy may help preserve residual beta cell mass in new-onset T1D patients. Whether this approach could prevent T1D onset in at-risk individuals is an open question and warrants future investigation.

## Diabetic Complications Indicating Islet Cell Replacement

Type 1 diabetes often results in large swings in blood glucose levels outside the normal physiologic range of 70–110 mg/dl. Studies of 50-year Joslin Medalists indicate that individuals with T1D can live for many decades with minimal or no diabetic complications (2, 3). In addition, recent advances in fast-acting synthetic insulin analogs, continuous glucose level monitoring technology, and early attempts at developing pump-like systems to deliver glucagon suggest that individuals with T1D would continue to see improvements in diabetes management and therefore in quality of life. However, even with adequate clinical control of blood sugar levels, long-term diabetic complications can develop in individuals with T1D. In addition, despite the technical and clinical advances noted above, some individuals with T1D nonetheless have labile blood glucose level control and are susceptible to severe and life-threatening disease-related complications. These chronic complications can affect essentially every organ system and are particularly pronounced in the microvasculature. Diabetes, T1D and T2D combined, is the leading cause of adult blindness [diabetic retinopathy (135)] and end-stage renal failure [diabetic nephropathy (136)], as well as a leading cause of lower-leg amputations [diabetic peripheral neuropathy (137)] and heart disease [diabetic cardiomyopathy (138, 139)]. Perhaps the most debilitating diabetic complication is hypoglycemic unawareness. This occurs when an individual with T1D is not aware their blood glucose levels are dangerously low (<50 mg/dl). This condition can result in seizures, diabetic coma, and, in the most severe cases, death. The development of hypoglycemia unawareness is thought to result from frequent, severe swings in blood glucose levels in some long-term T1D patients. Why hypoglycemia unawareness develops in some individuals but not others with long-term T1D is an open question. One possibility is that, over time, some T1D patients develop autoreactivity against glucagon-producing alpha cells. Glucagon-reactive CD8 T cells have been identified in NOD mice (140); therefore we speculate that some individuals with T1D may develop autoimmunity against alpha cells over time. Glucagon acts in opposition to insulin, promoting glycogen breakdown in the liver and therefore promoting increased blood glucose levels. If glucagon-derived peptides are associated with inflammation and cell death within the pancreas, existing autoreactive T cells could become primed in pancreas-draining lymph nodes, proliferate, and mediate destruction of glucagon-producing cells. In fact, there is emerging evidence that a small proportion of T1D patients develop antiglucagon antibodies (140). Another possibility is that destruction of autonomic innervation within pancreatic islets (141) leads to impaired communication with the hypothalamus, so that glucagon is not produced when signals are present based on blood glucose levels. If autonomic innervation of pancreatic islets is perturbed in individuals with T1D, the consequence could be a breakdown in communication with the hypothalamus. Interestingly, some T1D but not T2D subjects develop autoantibodies against the neuroendocrine protein tetraspanin7 from sympthatheic nerves within islets (142). In some patients with severe hypoglycemia, both of these scenarios, and others, could lead to impaired glucagon responses to hypoglycemia. Whole pancreas and isolated pancreatic islet transplantation are options to restore blood glucose level homeostasis for individuals with hypoglycemia unawareness. There are two potential sources of pancreatic beta cells for islet replacement, cadaveric (deceased) donors and iPS-derived beta cells (143), both of which are discussed below.

## Islet Graft Alloimmunity

### Islet Replacement Strategies

The current clinical strategy to replace the lost beta cell function is through whole pancreas or isolated pancreatic islet transplantation from genetically unrelated cadaveric donors. Because donors are limited, currently only T1D patients with hypoglycemia unawareness are considered for transplantation. This has created great interest in cell culture methods to produce large quantities of insulin-producing cells for transplantation. After more than 10 years of development, the Melton laboratory became the first group to develop a reproducible protocol for iPS conversion to insulin-producing beta cells (143), quickly followed by several other groups (144–146). However, these methods are not yet suitable for large-scale production of patient-specific iPS-beta cells for transplantation studies since individuals require several hundred thousand individual pancreatic islets. Furthermore, a critical limiting factor of a “universal donor” beta cell line is conventional transplant recognition, described below. In addition, unlike whole pancreas or isolated islet transplantation, iPS-beta cells do not replace the lost alpha cell function. Until these challenges are addressed, transplantation from a cadaveric donor will likely remain the preferred approach in combination with immune suppression (147, 148). A recent phase III clinical trial demonstrated improved glycemic control in islet transplant recipients following multisite standardized processing protocols (149, 150). As less beta cell-toxic immune suppression treatments are developed, we can expect transplant function and long-term survival to continue to improve. In the absence of these treatments, transplanted beta cells in autoimmune recipient patients would be subject to at least two categories of T cell responses: (a) autoimmune (islet-specific) responses by T cells (151, 152), and (b) conventional anti-transplant-reactive T cell responses. However, current immune suppression treatments do not promote immune tolerance as described above, must be continued indefinitely after transplantation, and can render the transplant recipient vulnerable to cancer and infectious agents. Therefore, transplant-specific tolerance-promoting treatments are a highly sought after goal in the islet transplantation field.

An alternative to replacing the lost beta cell mass would be to stimulate beta cell regeneration. Beta cell regeneration is based on the premise that if autoreactive T cells are removed or inhibited, existing beta cells could proliferate, alpha cells could convert into beta cells, or islet-resident stem cell populations could proliferate and differentiate into beta cells. There is little experimental evidence to support these suppositions to date. Beta cells are exceptionally metabolically active, continuously producing insulin secretory granules. The less beta cell mass is available to produce insulin, the higher the metabolic stress is on each individual islet. Therefore, the ability to regenerate beta cells from existing beta cells could be a significant hurdle. Another theoretical option to replace lost beta cell mass is to promote trans-differentiation of existing alpha cells into beta cells. Recent evidence from Kim’s laboratory at Stanford suggests that alpha cell conversion to beta cells may be feasible (153). However, even if beta cell replacement, alpha cell trans-differentiation, or beta cell regeneration succeed, these strategies do not address the deficiency in alpha cell glucagon production, which precipitates hypoglycemia unawareness, and as such do not represent a complete treatment for this life-threatening diabetic complication on its own. Therefore, whole islet transplantation will remain the clinical standard-of-care over beta cell replacement until these concerns can be fully addressed.

### Concurrent Autoimmune and Alloimmune Pathogenesis

There are two separate immune recognition pathways leading to the destruction of transplanted beta cells in the autoimmune recipient. As mentioned above, the first is autoimmunity due to antigen-specific memory T cells. Regardless of the source of beta cells transplanted into an individual with T1D, autoimmune T cells would target cells producing insulin and must be inhibited or removed to facilitate long-term transplant function (154). In contrast, autoreactive T cell targeting of a kidney transplant in a diabetic individual would not likely occur, because there would be no pre-existing kidney-specific memory T cells (154). Alloimmunity is the second major concern leading to the destruction of transplanted beta cells. Transplant-reactive or alloreactive T cell responses can target the genetic differences between the transplant donor and recipient (155). This category of immune response occurs against any organ or tissue transplant, in any individual, regardless of autoimmune disease status (156). Importantly, these transplant-specific responses focus primarily on the HLA molecule of the human transplant or MHC in mouse. HLA molecules are the most polymorphic loci in the human genome, and each individual expresses multiple alleles of both class I and class II HLA (154–156). All the genetic differences in both alleles are potential antigens and could be targeted by T cells in transplant recipients. The differences in HLA class I are targeted by recipient CD8 T cells, and the differences in HLA class II are targeted by recipient CD4 T cells (156). Ironically, genetic diversity in HLA promotes diverse T cell responses to the same pathogen in different individuals, but unfortunately these genetic differences also promote strong T cell responses against any transplanted organ or tissue. In this section, we describe transplant recognition and alloimmunity separately from autoimmunity.

### Transplant Recognition: Direct and Indirect Pathways

Donor-derived MHC (or HLA) molecules are the most prevalent transplant-derived antigen seen by the immune system of a transplant recipient. Transplant recipient T cells can interact with donor MHC molecules in two ways termed direct and indirect recognition (157). Direct allorecognition results from T cell interaction with donor MHC (plus some peptide loaded in MHC), whereas indirect allorecognition results from T cell interactions with recipient MHC (plus peptide derived from donor MHC, or any other transplant-derived protein). It is estimated that 1–10% of CD8 T cells or CD4 T cells will spontaneously respond to allogeneic MHC I or MHC II, respectively [reviewed in Ref. (158)]. In contrast, we hypothesize that the indirect precursor frequency is even smaller. In support of this hypothesis, recent evidence indicates that only 10% of allograft-reactive CD4 T cells in a mouse model of cardiac allograft rejection are indirect, while the remaining 90% are direct alloreactive CD4 T cells (159). Due to the higher precursor frequency for direct allorecognition than indirect allorecognition [reviewed in Ref. (157)], immune suppression protocols appear to hold direct alloreactivity in check. However, indirect recognition, which leads to antibody formation, CD4 T cell reactivity, and complement activation, is not completely inhibited using current immune suppression treatment regiments, as shown by complement deposition and antibody formation in chronic rejection models (160).

Importantly, both CD4 and CD8 T cells in the recipient can interact with donor MHC through either the direct or indirect pathway. The frequency and physiologic relevance of direct and indirect allorecognition varies with the nature of the transplanted organ or tissue. For islet allograft recognition, donor MHC class I and direct interaction with recipient CD8 T cells is a high-frequency event, because all cells in the graft express MHC class I. Since beta cells do not express MHC class II at baseline (161), direct recognition via CD4 T cells may not be as high frequency of an event. However, recent evidence suggests that beta cells may express MHC class II following T cell infiltration (161), which suggests that direct alloreactive CD4 T cells may be critical for anti-islet allograft responses. In contrast, indirect allorecognition by CD8 T cells must be therapeutically addressed to prevent islet allograft rejection (see below discussion of CD154 blockade therapy). Table 2 summarizes the roles of direct and indirect CD4 and CD8 T cells in islet allograft rejection in the NOD mouse model.

**Table 2:** Islet allograft recognition pathways and likely players in rejection in autoimmune diabetic recipients.

Direct or indirect	T cells	Target	Precursor frequency in recipients	Fold expansion posttransplant	Sufficient for rejection?	Required for rejection?	Reference
Direct	CD4 T cells	Donor MHC II + transplant-derived peptide	0.1–10% versus individual donor MHC	10–100	Yes	No	(162–164)
Direct	CD8 T cells	Donor MHC I + transplant-derived peptide	0.1–10% versus individual donor MHC	10–100	Yes	No	(162–164)
Indirect	CD4 T cells	Donor-derived peptide loaded in recipient MHC II	Less than 1 in 1,000,000	>100	Yes	Appears likely	(162–164)
Indirect	CD8 T cells	Donor-derived peptide loaded in recipient MHC I	Less than 1 in 1,000,000	>100	Yes	Appears not	(162–164)

### Islet Allograft Tolerance in Non-Autoimmune Diabetic Mice

Unfortunately, islet transplants are subject to both autoimmune disease recurrence and allograft recognition in T1D mice and humans. To remove autoimmunity as a confounding variable from islet transplant tolerance studies, several labs have made use of the free radical generator streptozotocin (STZ) (165–167). STZ induces diabetes due to the relative lack of free radical scavenging enzymes expressed in pancreatic beta cells relative to other cell types (168). Following induction of diabetes with STZ, mice can be transplanted with allogeneic (MHC-disparate) pancreatic islets and treated with candidate transplant tolerance-promoting therapies. In experiments using non-autoimmune diabetic mice, untreated recipients serve as control groups to determine time to normal allograft rejection.

Multiple different general immune suppressive therapies have been tested in preclinical mouse models and are used clinically (168). These therapies can include anti-CD3, antithymocyte globulin, calcineurin inhibitors, mTOR inhibitors, tacrolimus, or mycophenolate mofetil (169). Interestingly, one of the tolerance-promoting protocols, which reversed diabetes, ECDI-coupled splenocytes, can also promote islet allograft tolerance in non-autoimmune mice (170). Of particular interest, monoclonal antibodies to block T cell co-stimulation (or signal 2) have been tested by several groups (165, 171). For example, short-term monoclonal antibody therapy directed against the T cell-expressed co-stimulation molecule CD154 (CD40L) has been shown by several groups (165, 171) to induce long-term (>100 days) islet allograft tolerance across full MHC mismatch donor/recipient pairs (e.g., BALB/c islets transplanted into STZ-treated B6 male mice). This tolerance resides in the CD4 T cell compartment and can be transferred from treated and tolerant mice to naive mice (165). It is controversial whether this therapy induces allo-specific regulatory T cells *de novo* [suggested by Ferrer et al. (172)] or inhibits reactivity of naive alloreactive CD8 T cells through killing mediated by NK cells (173), or if these effects are simultaneous. In addition, the combination of anti-CD154 antibody with other therapies has been highly efficacious, in particular LFA-1 blockade. LFA-1 (CD11a) is an adhesion molecule expressed on most leukocytes, in particular on neutrophils, macrophages, and activated T cells. LFA-1 inhibition appears to delay and/or prevent islet allograft rejection as a single therapy. Similar to anti-CD154-induced transplant tolerance, uniform (100% of mice), long-term (>100 days) tolerance induced by the combination therapy of LFA-1 blockade and CD154 blockade resided in the CD4 T cell compartment and was serially transferable to multiple islet allograft recipients (165). In summary, STZ-induced diabetes represents a useful, non-autoimmune model system to test candidate islet allograft tolerance-promoting therapies. However, the end goal is to induce islet tolerance in autoimmune recipients, such as the NOD mouse.

In islet transplantation studies, “indirect” (recipient MHC-restricted) alloreactive CD4 T cells are key perpetrators of islet allograft rejection (174). As such, we hypothesize that co-transfer of islet antigen-specific Tregs at the time of islet transplantation would inhibit alloreactive T cell responses. Indeed, immune tolerance to antigen-presenting cell-depleted islet allografts in non-autoimmune mice requires CD4 T cells in transplant recipient mice (175). An alternative approach is to promote expression of T cell inhibitory receptor ligands on beta cells prior to transplantation (Figure 1). One example of this approach is beta cell expression of Fas ligand, which when combined with the immune suppressive drug rapamycin generated Tregs in recipient mice (176). Another example of this approach is a recent report which demonstrated that enforced beta cell-intrinsic PD-L1 and CTLA4 expression significantly delayed islet allograft rejection in NOD mice (177). In conclusion, whether autoimmunity or alloimmunity drives islet transplant rejection, generation, or adoptive transfer of Tregs or pre-arming transplanted beta cells with co-inhibitory molecules represent two distinct strategies to protect beta cells.

### Potential Role for Regulatory CD4 T Cells in the Autoimmune Recipient of an Islet Allograft

Importantly, regulatory CD4 Foxp3^+^ T cells engage peptides through the indirect antigen recognition pathway. Therefore, therapies that promote the development of transplant-specific Tregs are highly desirable. One long-term goal of the islet transplantation and autoimmunity field is to either deplete “indirect” autoreactive CD4 T cells or re-educate these CD4 T cells to become Foxp3^+^ regulatory CD4 T cells, while also generating additional “indirect” Tregs specific for transplant-derived antigens. Based on the above considerations for beta cell MHC II expression in the inflamed transplant recipient, we hypothesize that regulatory CD4 T cells specific for donor MHC II would prolong islet allograft survival. In addition, we hypothesize that conventional self-reactive and “indirect” CD4 T cells, which recognize autoantigens through the transplant recipient’s MHC class II molecule, would prolong graft survival. In combination, we speculate that adoptive transfer of both autoantigen-specific “indirect” Tregs as well as transplant MHC II-specific “direct” Tregs would synergize to significantly prolong islet allograft survival in autoimmune recipients.

### Failure of Islet Transplant Tolerance in the NOD Mouse

Laboratories at the Barbara Davis Center (31), Vanderbilt (178), Harvard (179), University of Massachusetts (180), University of North Carolina (181), the University of Miami (182), and the St. Vincent’s Institute in Melbourne (78) have utilized the NOD mouse as a model system to study both autoimmune disease recurrence (rejection of NOD-background islets) or islet allograft rejection (rejection of islet from genetically unrelated donor strains including B6, C3H). Due to its autoimmune disease status, the diabetic NOD female islet transplant recipient is a difficult, but clinically relevant model to test islet transplant tolerance-promoting therapies. Several studies have demonstrated the requirement for both CD4 T cells and CD8 T cells in diabetes recurrence in NOD mice (183, 184). Less data are available in the islet allograft scenario in NOD mice. Due to the sheer number of pancreatic islets required to reverse hyperglycemia and rapid T cell-mediated transplant rejection, diabetic female NOD mice are not frequently used to test transplant tolerance-promoting therapies.

The NOD mouse is an extremely stringent model to test transplant tolerance-promoting therapies. There are vanishingly few examples of long-term transplant tolerance in NOD mice. In particular, the combination of CD154 and LFA-1 in B6 mice resulted in long-term tolerance (180, 185). It is controversial whether this stringency results from resistance to therapeutic intervention in the autoimmune primed/memory T cell compartment, the alloreactive T cell response in NOD mice, or both. Mouse models and human clinical reports have suggested that autoimmune T cells are less susceptible to conventional immunosuppression (151, 185). In addition and in parallel, data from NOD mice support the existence of an accelerated and therapy-resistant anti-allograft T cell response (162). Additional studies in the Bio Breeder rat further suggested that autoimmune T cells are strongly impervious to tolerance-promoting therapy in this animal model of T1D, whereas the anti-allograft response can be made tolerant (186–189). These differences between models, and a lack of peptide-MHC II reagents to separately track both autoreactive and alloreactive CD4 T cells in the same transplant recipient mouse, lead to a lack of consensus in the field and an incomplete understanding of auto- and allo-T cell tolerance, in particular when both immune responses occur simultaneously.

While global immune suppressive treatments promote survival of transplanted beta cells [with the exception of calcineurin inhibitors, which are toxic to beta cells (190)], it is challenging to interpret effects of immune-modulatory therapies on specific T cell populations. Clinically, in the autoimmune recipient of pancreatic islets, there are at least two concurrent immune responses. As such, a major limiting factor in this analysis is the quality and availability of reagents to reliably and separately track autoreactive and alloreactive T cell responses in human patients. Lack of validated reagents to monitor these responses longitudinally in clinical samples presents a major challenge to interpret therapeutic effects on recurrent autoimmunity versus anti-allograft responses. Lack of reagents to separately assess these two categories of T cell responses in the NOD mouse prevents the development of reagents to preferentially influence either category of T cell response in the preclinical or clinical setting.

## Concluding Remarks

To prevent diabetes onset in the NOD mouse or at-risk human patients, several goals must be achieved. The genetics of T1D risks are well established, but the field lacks a comprehensive panel of peptide-HLA II tetramers to specifically track disease-associated CD4 T cell populations. Several groups (191–193), including our own (194), are working to fill this gap. Reagents to track key pathogenic CD4 T cells, perhaps including hybrid peptide-specific or DRiP-specific CD4 and CD8 T cells, are being developed and validated for clinical use. In addition, predictive biomarkers to measure not only the presence of these autoreactive T cells but also their activation status should be a focus of attention. Real-time monitoring of the activation status of rate-limiting autoreactive T cells is required to measure the efficacy of any tolerance-promoting therapy. Finally, to establish beta cell protection, measurements of beta cell function are required, in combination with assessment of autoreactive T cell biology. Non-invasive imaging methods represent one option (195, 196), but require specialized imaging technology and may not have sufficient sensitivity. More recently, methods such as high-sensitivity C-peptide assays (46, 197) and quantification of demethylated insulin DNA in the circulation (198, 199) could accomplish this beta cell health surveillance goal.

Despite our understanding of diabetes pathogenesis and ever-improving clinical care for individuals with T1D, some individuals develop debilitating diabetic complications that necessitate whole pancreas or isolated islet transplantation. In the autoimmune recipient, two categories of T cell responses must be prevented or inhibited to promote long-term transplant function. Both memory autoimmune T cell responses and nascent T cell responses against polymorphic MHC molecules occur after pancreas, islets, or iPS-beta cell replacement in T1D individuals. Therefore, a thorough understanding of not only autoimmune pathogenesis but also transplant recognition is required to develop methods to protect transplanted beta cells in autoimmune individuals. Intriguingly, Foxp3^+^CD4 regulatory T cells may represent a path toward developing antigen-specific tolerance in both autoimmunity and transplant recognition. As such, immunotherapies that promote the development of regulatory CD4 T cells in both autoimmune models and transplantation models are highly desirable.

Multiple challenges remain to achieve the elusive goal of preventing islet transplant rejection in autoimmune recipients. Chief among these is to more specifically define the roles of CD4 and CD8 T cells and to determine whether autoimmunity or alloimmunity represents the higher barrier to beta cell transplant survival. Additional challenges to establishing islet allograft tolerance in the autoimmune recipient include (a) determining whether removing MHC from islet allografts would delay transplant rejection, (b) investigating if there is overlap of autoimmunity and alloreactivity on the individual T cell level, as has been shown for viral memory and transplant rejection (200, 201), (c) understanding if an MHC-matched “universal donor” beta cell line would avoid alloimmune T cell responses, (d) determining if beta cells can be induced to express proteins that would protect a transplant, and (e) developing reagents to track “direct” alloreactivity (against donor MHC). We and others are working to determine answers to these and other critical questions. With coordinated work by many dedicated individuals, we anticipate further advancements in our understanding of autoimmune pathogenesis, beta cell biology, and transplant recognition.

## Author Contributions

ALB, TM, and BTF wrote and edited the review article. TM and BTF created the model figure.

## Conflict of Interest Statement

The authors declare that the research was conducted in the absence of any commercial or financial relationships that could be construed as a potential conflict of interest.

## References

[1] Atkinson MA Eisenbarth GS Michels AW. Type 1 diabetes. Lancet (2014) 383(9911):69–82. 10.1016/S0140-6736(13)60591-7 23890997 PMC4380133

[2] Keenan HA Sun JK Levine J Doria A Aiello LP Eisenbarth G Residual insulin production and pancreatic β-cell turnover after 50 years of diabetes: Joslin Medalist Study. Diabetes (2010) 59(11):2846–53. 10.2337/db10-0676 20699420 PMC2963543

[3] Sun JK Keenan HA Cavallerano JD Asztalos BF Schaefer EJ Sell DR Protection from retinopathy and other complications in patients with type 1 diabetes of extreme duration: the Joslin 50-year Medalist Study. Diabetes Care (2011) 34(4):968–74. 10.2337/dc10-1675 21447665 PMC3064059

[4] Rodriguez-Calvo T Ekwall O Amirian N Zapardiel-Gonzalo J von Herrath MG. Increased immune cell infiltration of the exocrine pancreas: a possible contribution to the pathogenesis of type 1 diabetes. Diabetes (2014) 63(11):3880–90. 10.2337/db14-0549 24947367 PMC4207385

[5] Pathiraja V Kuehlich JP Campbell PD Krishnamurthy B Loudovaris T Coates PT Proinsulin-specific, HLA-DQ8, and HLA-DQ8-transdimer-restricted CD4+ T cells infiltrate islets in type 1 diabetes. Diabetes (2015) 64(1):172–82. 10.2337/db14-0858 25157096

[6] Soeldner JS Tuttleman M Srikanta S Ganda OP Eisenbarth GS Insulin-dependent diabetes mellitus and autoimmunity: islet-cell autoantibodies, insulin autoantibodies, and beta-cell failure. N Engl J Med (1985) 313(14):893–4. 10.1056/NEJM198510033131417 3897867

[7] Eisenbarth GS Type I diabetes mellitus. A chronic autoimmune disease. N Engl J Med (1986) 314(21):1360–8. 10.1056/NEJM198605223142106 3517648

[8] Zhang L Gianani R Nakayama M Liu E Kobayashi M Baschal E Type 1 diabetes: chronic progressive autoimmune disease. Novartis Found Symp (2008) 292:85–94; discussion 94–8, 122–9, 202–3. 10.1002/9780470697405.ch7 19203094

[9] Sutherland DE Goetz FC Sibley RK. Recurrence of disease in pancreas transplants. Diabetes (1989) 38(Suppl 1):85–7. 10.2337/diab.38.1.S85 2642862

[10] Coppieters KT Dotta F Amirian N Campbell PD Kay TW Atkinson MA Demonstration of islet-autoreactive CD8 T cells in insulitic lesions from recent onset and long-term type 1 diabetes patients. J Exp Med (2012) 209(1):51–60. 10.1084/jem.20111187 22213807 PMC3260877

[11] Sarikonda G Pettus J Phatak S Sachithanantham S Miller JF Wesley JD CD8 T-cell reactivity to islet antigens is unique to type 1 while CD4 T-cell reactivity exists in both type 1 and type 2 diabetes. J Autoimmun (2014) 50:77–82. 10.1016/j.jaut.2013.12.003 24387802

[12] Rodriguez-Calvo T Suwandi JS Amirian N Zapardiel-Gonzalo J Anquetil F Sabouri S Heterogeneity and lobularity of pancreatic pathology in type 1 diabetes during the prediabetic phase. J Histochem Cytochem (2015) 63(8):626–36. 10.1369/0022155415576543 26216138 PMC4530397

[13] Atkinson MA. The pathogenesis and natural history of type 1 diabetes. Cold Spring Harb Perspect Med (2012) 2(11). 10.1101/cshperspect.a007641 23125199 PMC3543105

[14] Pietropaolo M Towns R Eisenbarth GS. Humoral autoimmunity in type 1 diabetes: prediction, significance, and detection of distinct disease subtypes. Cold Spring Harb Perspect Med (2012) 2(10). 10.1101/cshperspect.a012831 23028135 PMC3475400

[15] Pociot F Lernmark A. Genetic risk factors for type 1 diabetes. Lancet (2016) 387(10035):2331–9. 10.1016/S0140-6736(16)30582-7 27302272

[16] Thayer TC Delano M Liu C Chen J Padgett LE Tse HM Superoxide production by macrophages and T cells is critical for the induction of autoreactivity and type 1 diabetes. Diabetes (2011) 60(8):2144–51. 10.2337/db10-1222 21715554 PMC3142064

[17] Padgett LE Anderson B Liu C Ganini D Mason RP Piganelli JD Loss of NOX-derived superoxide exacerbates diabetogenic CD4 T-cell effector responses in type 1 diabetes. Diabetes (2015) 64(12):4171–83. 10.2337/db15-0546 26269022 PMC4657579

[18] Makino S Kunimoto K Muraoka Y Mizushima Y Katagiri K Tochino Y. Breeding of a non-obese, diabetic strain of mice. Jikken Dobutsu (1980) 29(1):1–13. 6995140 10.1538/expanim1978.29.1_1

[19] Anderson MS Bluestone JA. The NOD mouse: a model of immune dysregulation. Annu Rev Immunol (2005) 23:447–85. 10.1146/annurev.immunol.23.021704.115643 15771578

[20] Mullen Y. Development of the nonobese diabetic mouse and contribution of animal models for understanding type 1 diabetes. Pancreas (2017) 46(4):455–66. 10.1097/MPA.0000000000000828 28291161 PMC5359790

[21] von Herrath MG Homann D Gairin JE Oldstone MB Pathogenesis and treatment of virus-induced autoimmune diabetes: novel insights gained from the RIP-LCMV transgenic mouse model. Biochem Soc Trans (1997) 25(2):630–5. 10.1042/bst0250630 9191170

[22] Kurts C Heath WR Carbone FR Kosaka H Miller JF Cross-presentation of self antigens to CD8+ T cells: the balance between tolerance and autoimmunity. Novartis Found Symp (1998) 215:172–81; discussion 181–90. 9760579 10.1002/9780470515525.ch13

[23] Apostolou I Von Boehmer H The TCR-HA, INS-HA transgenic model of autoimmune diabetes: limitations and expectations. J Autoimmun (2004) 22(2):111–4. 10.1016/j.jaut.2003.10.005 14987738

[24] Van Belle TL Taylor P von Herrath MG. Mouse models for type 1 diabetes. Drug Discov Today Dis Models (2009) 6(2):41–5. 10.1016/j.ddmod.2009.03.008 20407588 PMC2855847

[25] Mora C Wong FS Chang CH Flavell RA Pancreatic infiltration but not diabetes occurs in the relative absence of MHC class II-restricted CD4 T cells: studies using NOD/CIITA-deficient mice. J Immunol (1999) 162(8):4576–88. 10201997

[26] Serreze DV Leiter EH Christianson GJ Greiner D Roopenian DC. Major histocompatibility complex class I-deficient NOD-B2mnull mice are diabetes and insulitis resistant. Diabetes (1994) 43(3):505–9. 10.2337/diabetes.43.3.505 8314025

[27] Yang M Charlton B Gautam AM. Development of insulitis and diabetes in B cell-deficient NOD mice. J Autoimmun (1997) 10(3):257–60. 10.1006/jaut.1997.0128 9218752

[28] Noorchashm H Noorchashm N Kern J Rostami SY Barker CF Naji A. B-cells are required for the initiation of insulitis and sialitis in nonobese diabetic mice. Diabetes (1997) 46(6):941–6. 10.2337/diabetes.46.6.941 9166663

[29] Zucchelli S Holler P Yamagata T Roy M Benoist C Mathis D. Defective central tolerance induction in NOD mice: genomics and genetics. Immunity (2005) 22(3):385–96. 10.1016/j.immuni.2005.01.015 15780994

[30] Wicker LS Appel MC Dotta F Pressey A Miller BJ DeLarato NH Autoimmune syndromes in major histocompatibility complex (MHC) congenic strains of nonobese diabetic (NOD) mice. The NOD MHC is dominant for insulitis and cyclophosphamide-induced diabetes. J Exp Med (1992) 176(1):67–77. 10.1084/jem.176.1.67 1613467 PMC2119272

[31] Wang Y Hao L Gill RG Lafferty KJ. Autoimmune diabetes in NOD mouse is L3T4 T-lymphocyte dependent. Diabetes (1987) 36(4):535–8. 10.2337/diabetes.36.4.535 3102301

[32] Shizuru JA Taylor-Edwards C Banks BA Gregory AK Fathman CG. Immunotherapy of the nonobese diabetic mouse: treatment with an antibody to T-helper lymphocytes. Science (1988) 240(4852):659–62. 10.1126/science.2966437 2966437

[33] Hutchings P O’Reilly L Parish NM Waldmann H Cooke A. The use of a non-depleting anti-CD4 monoclonal antibody to re-establish tolerance to beta cells in NOD mice. Eur J Immunol (1992) 22(7):1913–8. 10.1002/eji.1830220735 1623929

[34] Katz JD Benoist C Mathis D. T helper cell subsets in insulin-dependent diabetes. Science (1995) 268(5214):1185–8. 10.1126/science.7761837 7761837

[35] Arif S Tree TI Astill TP Tremble JM Bishop AJ Dayan CM Autoreactive T cell responses show proinflammatory polarization in diabetes but a regulatory phenotype in health. J Clin Invest (2004) 113(3):451–63. 10.1172/JCI19585 14755342 PMC324541

[36] Michels AW Landry LG McDaniel KA Yu L Campbell-Thompson M Kwok WW Islet-derived CD4 T cells targeting proinsulin in human autoimmune diabetes. Diabetes (2017) 66(3):722–34. 10.2337/db16-1025 27920090 PMC5319719

[37] Gómez-Touriño I Simón-Vázquez R Alonso-Lorenzo J Arif S Calviño-Sampedro C González-Fernández Á Characterization of the autoimmune response against the nerve tissue S100beta in patients with type 1 diabetes. Clin Exp Immunol (2015) 180(2):207–17. 10.1111/cei.12572 25516468 PMC4408155

[38] Arif S Moore F Marks K Bouckenooghe T Dayan CM Planas R Peripheral and islet interleukin-17 pathway activation characterizes human autoimmune diabetes and promotes cytokine-mediated beta-cell death. Diabetes (2011) 60(8):2112–9. 10.2337/db10-1643 21659501 PMC3142078

[39] Bellemore SM Nikoopour E Schwartz JA Krougly O Lee-Chan E Singh B. Preventative role of interleukin-17 producing regulatory T helper type 17 (Treg 17) cells in type 1 diabetes in non-obese diabetic mice. Clin Exp Immunol (2015) 182(3):261–9. 10.1111/cei.12691 26250153 PMC4636888

[40] Babon JA DeNicola ME Blodgett DM Crèvecoeur I Buttrick TS Maehr R Analysis of self-antigen specificity of islet-infiltrating T cells from human donors with type 1 diabetes. Nat Med (2016) 22(12):1482–7. 10.1038/nm.4203 27798614 PMC5140746

[41] Trivedi P Graham KL Krishnamurthy B Fynch S Slattery RM Kay TW Perforin facilitates beta cell killing and regulates autoreactive CD8+ T-cell responses to antigen in mouse models of type 1 diabetes. Immunol Cell Biol (2016) 94(4):334–41. 10.1038/icb.2015.89 26446877

[42] DiLorenzo TP Graser RT Ono T Christianson GJ Chapman HD Roopenian DC Major histocompatibility complex class I-restricted T cells are required for all but the end stages of diabetes development in nonobese diabetic mice and use a prevalent T cell receptor alpha chain gene rearrangement. Proc Natl Acad Sci U S A (1998) 95(21):12538–43. 10.1073/pnas.95.21.12538 9770521 PMC22866

[43] Hamilton-Williams EE Palmer SE Charlton B Slattery RM. Beta cell MHC class I is a late requirement for diabetes. Proc Natl Acad Sci U S A (2003) 100(11):6688–93. 10.1073/pnas.1131954100 12750472 PMC164508

[44] Wong FS Karttunen J Dumont C Wen L Visintin I Pilip IM Identification of an MHC class I-restricted autoantigen in type 1 diabetes by screening an organ-specific cDNA library. Nat Med (1999) 5(9):1026–31. 10.1038/12465 10470079

[45] Nakayama M Abiru N Moriyama H Babaya N Liu E Miao D Prime role for an insulin epitope in the development of type 1 diabetes in NOD mice. Nature (2005) 435(7039):220–3. 10.1038/nature03523 15889095 PMC1364531

[46] Roep BO Solvason N Gottlieb PA Abreu JRF Harrison LC Eisenbarth GS Plasmid-encoded proinsulin preserves C-peptide while specifically reducing proinsulin-specific CD8(+) T cells in type 1 diabetes. Sci Transl Med (2013) 5(191):191ra82 10.1126/scitranslmed.3006103 PMC4516024 23803704

[47] Suk K Kim S Kim YH Kim KA Chang I Yagita H IFN-gamma/TNF-alpha synergism as the final effector in autoimmune diabetes: a key role for STAT1/IFN regulatory factor-1 pathway in pancreatic beta cell death. J Immunol (2001) 166(7):4481–9. 10.4049/jimmunol.166.7.4481 11254704

[48] Chen J Grieshaber S Mathews CE. Methods to assess beta cell death mediated by cytotoxic T lymphocytes. J Vis Exp (2011) 52. 10.3791/2724 21712795 PMC3217247

[49] Calderon B Suri A Pan XO Mills JC Unanue ER. IFN-gamma-dependent regulatory circuits in immune inflammation highlighted in diabetes. J Immunol (2008) 181(10):6964–74. 10.4049/jimmunol.181.10.6964 18981116 PMC2653256

[50] Bender C Christen S Scholich K Bayer M Pfeilschifter JM Hintermann E Islet-expressed CXCL10 promotes autoimmune destruction of islet isografts in mice with type 1 diabetes. Diabetes (2017) 66(1):113–26. 10.2337/db16-0547 27797910

[51] Sarkar SA Lee CE Victorino F Nguyen TT Walters JA Burrack A Expression and regulation of chemokines in murine and human type 1 diabetes. Diabetes (2012) 61(2):436–46. 10.2337/db11-0853 22210319 PMC3266427

[52] Calderon B Suri A Unanue ER. In CD4+ T-cell-induced diabetes, macrophages are the final effector cells that mediate islet beta-cell killing: studies from an acute model. Am J Pathol (2006) 169(6):2137–47. 10.2353/ajpath.2006.060539 17148676 PMC1762478

[53] Padgett LE Burg AR Lei W Tse HM. Loss of NADPH oxidase-derived superoxide skews macrophage phenotypes to delay type 1 diabetes. Diabetes (2015) 64(3):937–46. 10.2337/db14-0929 25288672 PMC4338593

[54] Nikolic T Geutskens SB van Rooijen N Drexhage HA Leenen PJ. Dendritic cells and macrophages are essential for the retention of lymphocytes in (peri)-insulitis of the nonobese diabetic mouse: a phagocyte depletion study. Lab Invest (2005) 85(4):487–501. 10.1038/labinvest.3700238 15654358

[55] Ferris ST Carrero JA Mohan JF Calderon B Murphy KM Unanue ER. A minor subset of Batf3-dependent antigen-presenting cells in islets of Langerhans is essential for the development of autoimmune diabetes. Immunity (2014) 41(4):657–69. 10.1016/j.immuni.2014.09.012 25367577 PMC4220295

[56] Serreze DV Fleming SA Chapman HD Richard SD Leiter EH Tisch RM. B lymphocytes are critical antigen-presenting cells for the initiation of T cell-mediated autoimmune diabetes in nonobese diabetic mice. J Immunol (1998) 161(8):3912–8. 9780157

[57] Ziegler AG Vardi P Ricker AT Hattori M Soeldner JS Eisenbarth GS. Radioassay determination of insulin autoantibodies in NOD mice. Correlation with increased risk of progression to overt diabetes. Diabetes (1989) 38(3):358–63. 10.2337/diab.38.3.358 2917700

[58] Yu L Robles DT Abiru N Kaur P Rewers M Kelemen K Early expression of antiinsulin autoantibodies of humans and the NOD mouse: evidence for early determination of subsequent diabetes. Proc Natl Acad Sci U S A (2000) 97(4):1701–6. 10.1073/pnas.040556697 10677521 PMC26499

[59] Melanitou E Devendra D Liu E Miao D Eisenbarth GS. Early and quantal (by litter) expression of insulin autoantibodies in the nonobese diabetic mice predict early diabetes onset. J Immunol (2004) 173(11):6603–10. 10.4049/jimmunol.173.11.6603 15557150

[60] Srikanta S Ganda OP Rabizadeh A Soeldner JS Eisenbarth GS. First-degree relatives of patients with type I diabetes mellitus. Islet-cell antibodies and abnormal insulin secretion. N Engl J Med (1985) 313(8):461–4. 10.1056/NEJM198508223130801 3894969

[61] Pöllänen PM Lempainen J Laine AP Toppari J Veijola R Vähäsalo P Characterisation of rapid progressors to type 1 diabetes among children with HLA-conferred disease susceptibility. Diabetologia (2017) 60(7):1284–93. 10.1007/s00125-017-4258-7 28364254

[62] Roep BO Peakman M Antigen targets of type 1 diabetes autoimmunity. Cold Spring Harb Perspect Med (2012) 2(4):a007781 10.1101/cshperspect.a007781 22474615 PMC3312399

[63] Wenzlau JM Juhl K Yu L Moua O Sarkar SA Gottlieb P The cation efflux transporter ZnT8 (Slc30A8) is a major autoantigen in human type 1 diabetes. Proc Natl Acad Sci U S A (2007) 104(43):17040–5. 10.1073/pnas.0705894104 17942684 PMC2040407

[64] Haskins K Portas M Bradley B Wegmann D Lafferty K. T-lymphocyte clone specific for pancreatic islet antigen. Diabetes (1988) 37(10):1444–8. 10.2337/diab.37.10.1444 2458291

[65] Haskins K Portas M Bergman B Lafferty K Bradley B. Pancreatic islet-specific T-cell clones from nonobese diabetic mice. Proc Natl Acad Sci U S A (1989) 86(20):8000–4. 10.1073/pnas.86.20.8000 2510155 PMC298201

[66] Wegmann DR Shehadeh N Lafferty KJ Norbury-Glaser M Gill RG Daniel D. Establishment of islet-specific T-cell lines and clones from islet isografts placed in spontaneously diabetic NOD mice. J Autoimmun (1993) 6(5):517–27. 10.1006/jaut.1993.1043 7902094

[67] Haskins K. Pathogenic T-cell clones in autoimmune diabetes: more lessons from the NOD mouse. Adv Immunol (2005) 87:123–62. 10.1016/S0065-2776(05)87004-X 16102573

[68] Tse HM Milton MJ Schreiner S Profozich JL Trucco M Piganelli JD. Disruption of innate-mediated proinflammatory cytokine and reactive oxygen species third signal leads to antigen-specific hyporesponsiveness. J Immunol (2007) 178(2):908–17. 10.4049/jimmunol.178.2.908 17202352

[69] Katz JD Wang B Haskins K Benoist C Mathis D. Following a diabetogenic T cell from genesis through pathogenesis. Cell (1993) 74(6):1089–100. 10.1016/0092-8674(93)90730-E 8402882

[70] Han B Serra P Amrani A Yamanouchi J Marée AF Edelstein-Keshet L Prevention of diabetes by manipulation of anti-IGRP autoimmunity: high efficiency of a low-affinity peptide. Nat Med (2005) 11(6):645–52. 10.1038/nm1250 15908957

[71] Stadinski BD Delong T Reisdorph N Reisdorph R Powell RL Armstrong M Chromogranin A is an autoantigen in type 1 diabetes. Nat Immunol (2010) 11(3):225–31. 10.1038/ni.1844 20139986 PMC3166626

[72] Delong T Baker RL Reisdorph N Reisdorph R Powell RL Armstrong M Islet amyloid polypeptide is a target antigen for diabetogenic CD4+ T cells. Diabetes (2011) 60(9):2325–30. 10.2337/db11-0288 21734016 PMC3161333

[73] Baker RL Delong T Barbour G Bradley B Nakayama M Haskins K. Cutting edge: CD4 T cells reactive to an islet amyloid polypeptide peptide accumulate in the pancreas and contribute to disease pathogenesis in nonobese diabetic mice. J Immunol (2013) 191(8):3990–4. 10.4049/jimmunol.1301480 24043895 PMC3815676

[74] Wenzlau JM Walter M Gardner TJ Frisch LM Yu L Eisenbarth GS Kinetics of the post-onset decline in zinc transporter 8 autoantibodies in type 1 diabetic human subjects. J Clin Endocrinol Metab (2010) 95(10):4712–9. 10.1210/jc.2010-0169 20610599 PMC3050104

[75] Dang M Rockell J Wagner R Wenzlau JM Yu L Hutton JC Human type 1 diabetes is associated with T cell autoimmunity to zinc transporter 8. J Immunol (2011) 186(10):6056–63. 10.4049/jimmunol.1003815 21471440 PMC3088514

[76] Prasad S Kohm AP McMahon JS Luo X Miller SD. Pathogenesis of NOD diabetes is initiated by reactivity to the insulin B chain 9-23 epitope and involves functional epitope spreading. J Autoimmun (2012) 39(4):347–53. 10.1016/j.jaut.2012.04.005 22647732 PMC3434243

[77] Fife BT Guleria I Gubbels Bupp M Eagar TN Tang Q Bour-Jordan H Insulin-induced remission in new-onset NOD mice is maintained by the PD-1-PD-L1 pathway. J Exp Med (2006) 203(12):2737–47. 10.1084/jem.20061577 17116737 PMC2118162

[78] Krishnamurthy B Dudek NL McKenzie MD Purcell AW Brooks AG Gellert S Responses against islet antigens in NOD mice are prevented by tolerance to proinsulin but not IGRP. J Clin Invest (2006) 116(12):3258–65. 10.1172/JCI29602 17143333 PMC1679712

[79] DiLorenzo TP. Multiple antigens versus single major antigen in type 1 diabetes: arguing for multiple antigens. Diabetes Metab Res Rev (2011) 27(8):778–83. 10.1002/dmrr.1251 22069259

[80] Jin N Wang Y Crawford F White J Marrack P Dai S N-terminal additions to the WE14 peptide of chromogranin A create strong autoantigen agonists in type 1 diabetes. Proc Natl Acad Sci U S A (2015) 112(43):13318–23. 10.1073/pnas.1517862112 26453556 PMC4629350

[81] Delong T Wiles TA Baker RL Bradley B Barbour G Reisdorph R Pathogenic CD4 T cells in type 1 diabetes recognize epitopes formed by peptide fusion. Science (2016) 351(6274):711–4. 10.1126/science.aad2791 26912858 PMC4884646

[82] Kracht MJ van Lummel M Nikolic T Joosten AM Laban S van der Slik AR Autoimmunity against a defective ribosomal insulin gene product in type 1 diabetes. Nat Med (2017) 23(4):501–7. 10.1038/nm.4289 28263308

[83] Zhang L Nakayama M Eisenbarth GS. Insulin as an autoantigen in NOD/human diabetes. Curr Opin Immunol (2008) 20(1):111–8. 10.1016/j.coi.2007.11.005 18178393 PMC2268871

[84] Kaufman DL Clare-Salzler M Tian J Forsthuber T Ting GS Robinson P Spontaneous loss of T-cell tolerance to glutamic acid decarboxylase in murine insulin-dependent diabetes. Nature (1993) 366(6450):69–72. 10.1038/366069a0 7694152 PMC8216222

[85] Atkinson MA Kaufman DL Newman D Tobin AJ Maclaren NK. Islet cell cytoplasmic autoantibody reactivity to glutamate decarboxylase in insulin-dependent diabetes. J Clin Invest (1993) 91(1):350–6. 10.1172/JCI116192 8423231 PMC330033

[86] Schloot NC Daniel D Norbury-Glaser M Wegmann DR. Peripheral T cell clones from NOD mice specific for GAD65 peptides: lack of islet responsiveness or diabetogenicity. J Autoimmun (1996) 9(3):357–63. 10.1006/jaut.1996.0048 8816971

[87] Videbaek N Harach S Phillips J Hutchings P Ozegbe P Michelsen BK An islet-homing NOD CD8+ cytotoxic T cell clone recognizes GAD65 and causes insulitis. J Autoimmun (2003) 20(2):97–109. 10.1016/S0896-8411(03)00003-9 12657523

[88] Ott PA Dittrich MT Herzog BA Guerkov R Gottlieb PA Putnam AL T cells recognize multiple GAD65 and proinsulin epitopes in human type 1 diabetes, suggesting determinant spreading. J Clin Immunol (2004) 24(4):327–39. 10.1023/B:JOCI.0000029120.77824.41 15163889

[89] Wenzlau JM Frisch LM Hutton JC Davidson HW. Mapping of conformational autoantibody epitopes in ZNT8. Diabetes Metab Res Rev (2011) 27(8):883–6. 10.1002/dmrr.1266 22069278 PMC3664522

[90] Énée É Kratzer R Arnoux JB Barilleau E Hamel Y Marchi C ZnT8 is a major CD8+ T cell-recognized autoantigen in pediatric type 1 diabetes. Diabetes (2012) 61(7):1779–84. 10.2337/db12-0071 22586580 PMC3379659

[91] Xu X Gu Y Bian L Shi Y Cai Y Chen Y Characterization of immune response to novel HLA-A2-restricted epitopes from zinc transporter 8 in type 1 diabetes. Vaccine (2016) 34(6):854–62. 10.1016/j.vaccine.2015.10.108 26541133

[92] Nayak DK Calderon B Vomund AN Unanue ER. ZnT8-reactive T cells are weakly pathogenic in NOD mice but can participate in diabetes under inflammatory conditions. Diabetes (2014) 63(10):3438–48. 10.2337/db13-1882 24812429 PMC4171664

[93] Kubosaki A Miura J Notkins AL IA-2 is not required for the development of diabetes in NOD mice. Diabetologia (2004) 47(1):149–50. 10.1007/s00125-003-1252-z 14614561

[94] Ouyang Q Standifer NE Qin H Gottlieb P Verchere CB Nepom GT Recognition of HLA class I-restricted beta-cell epitopes in type 1 diabetes. Diabetes (2006) 55(11):3068–74. 10.2337/db06-0065 17065344

[95] Zhao Z Miao D Michels A Steck A Dong F Rewers M A multiplex assay combining insulin, GAD, IA-2 and transglutaminase autoantibodies to facilitate screening for pre-type 1 diabetes and celiac disease. J Immunol Methods (2016) 430:28–32. 10.1016/j.jim.2016.01.011 26809048 PMC5851776

[96] Kawasaki E Hutton JC Eisenbarth GS. Molecular cloning and characterization of the human transmembrane protein tyrosine phosphatase homologue, phogrin, an autoantigen of type 1 diabetes. Biochem Biophys Res Commun (1996) 227(2):440–7. 10.1006/bbrc.1996.1526 8878534

[97] Kawasaki E Eisenbarth GS Wasmeier C Hutton JC. Autoantibodies to protein tyrosine phosphatase-like proteins in type I diabetes. Overlapping specificities to phogrin and ICA512/IA-2. Diabetes (1996) 45(10):1344–9. 10.2337/diab.45.10.1344 8826969

[98] Kelemen K Crawford ML Gill RG Hutton JC Wegmann D. Cellular immune response to phogrin in the NOD mouse: cloned T-cells cause destruction of islet transplants. Diabetes (1999) 48(8):1529–34. 10.2337/diabetes.48.8.1529 10426369

[99] Kelemen K Gottlieb PA Putnam AL Davidson HW Wegmann DR Hutton JC. HLA-DQ8-associated T cell responses to the diabetes autoantigen phogrin (IA-2 beta) in human prediabetes. J Immunol (2004) 172(6):3955–62. 10.4049/jimmunol.172.6.3955 15004204

[100] Karges W Hammond-McKibben D Gaedigk R Shibuya N Cheung R Dosch HM. Loss of self-tolerance to ICA69 in nonobese diabetic mice. Diabetes (1997) 46(10):1548–56. 10.2337/diabetes.46.10.1548 9313748

[101] Winer S Astsaturov I Gaedigk R Hammond-McKibben D Pilon M Song A ICA69(null) nonobese diabetic mice develop diabetes, but resist disease acceleration by cyclophosphamide. J Immunol (2002) 168(1):475–82. 10.4049/jimmunol.168.1.475 11751995

[102] Winer S Tsui H Lau A Song A Li X Cheung RK Autoimmune islet destruction in spontaneous type 1 diabetes is not beta-cell exclusive. Nat Med (2003) 9(2):198–205. 10.1038/nm818 12539039

[103] Chen W Bergerot I Elliott JF Harrison LC Abiru N Eisenbarth GS Evidence that a peptide spanning the B-C junction of proinsulin is an early autoantigen epitope in the pathogenesis of type 1 diabetes. J Immunol (2001) 167(9):4926–35. 10.4049/jimmunol.167.9.4926 11673498

[104] Gottlieb PA Delong T Baker RL Fitzgerald-Miller L Wagner R Cook G Chromogranin A is a T cell antigen in human type 1 diabetes. J Autoimmun (2014) 50:38–41. 10.1016/j.jaut.2013.10.003 24239002 PMC3995825

[105] Nikoopour E Krougly O Lee-Chan E Haeryfar SM Singh B. Detection of vasostatin-1-specific CD8(+) T cells in non-obese diabetic mice that contribute to diabetes pathogenesis. Clin Exp Immunol (2016) 185(3):292–300. 10.1111/cei.12811 27185276 PMC4991514

[106] Viret C Mahiddine K Baker RL Haskins K Guerder S. The T cell repertoire-diversifying enzyme TSSP contributes to thymic selection of diabetogenic CD4 T cell specificities reactive to ChgA and IAPP autoantigens. J Immunol (2015) 195(5):1964–73. 10.4049/jimmunol.1401683 26209627

[107] Wiles TA Delong T Baker RL Bradley B Barbour G Powell RL An insulin-IAPP hybrid peptide is an endogenous antigen for CD4 T cells in the non-obese diabetic mouse. J Autoimmun (2017) 78:11–8. 10.1016/j.jaut.2016.10.007 27802879 PMC5337175

[108] Yang J Danke NA Berger D Reichstetter S Reijonen H Greenbaum C Islet-specific glucose-6-phosphatase catalytic subunit-related protein-reactive CD4+ T cells in human subjects. J Immunol (2006) 176(5):2781–9. 10.4049/jimmunol.176.5.2781 16493034

[109] Ko HJ Chee J Sutherland RM Thomas HE Zhan Y Krishnamurthy B Functional cytotoxic T lymphocytes against IGRP206-214 predict diabetes in the non-obese diabetic mouse. Immunol Cell Biol (2014) 92(7):640–4. 10.1038/icb.2014.29 24777314

[110] Chentoufi AA Polychronakos C. Insulin expression levels in the thymus modulate insulin-specific autoreactive T-cell tolerance: the mechanism by which the IDDM2 locus may predispose to diabetes. Diabetes (2002) 51(5):1383–90. 10.2337/diabetes.51.5.1383 11978634

[111] Marrack P Kappler JW. Do MHCII-presented neoantigens drive type 1 diabetes and other autoimmune diseases? Cold Spring Harb Perspect Med (2012) 2(9):a007765. 10.1101/cshperspect.a007765 22951444 PMC3426820

[112] Lamhamedi-Cherradi SE Luan JJ Eloy L Fluteau G Bach JF Garchon HJ. Resistance of T-cells to apoptosis in autoimmune diabetic (NOD) mice is increased early in life and is associated with dysregulated expression of Bcl-x. Diabetologia (1998) 41(2):178–84. 10.1007/s001250050887 9498651

[113] Pugliese A Zeller M Fernandez A Jr Zalcberg LJ Bartlett RJ Ricordi C The insulin gene is transcribed in the human thymus and transcription levels correlated with allelic variation at the INS VNTR-IDDM2 susceptibility locus for type 1 diabetes. Nat Genet (1997) 15(3):293–7. 10.1038/ng0397-293 9054945

[114] Regnault B Osorio Y Fortea J Miao D Eisenbarth G Melanitou E. Early over expression of messenger RNA for multiple genes, including insulin, in the pancreatic lymph nodes of NOD mice is associated with islet autoimmunity. BMC Med Genomics (2009) 2:63. 10.1186/1755-8794-2-63 19799787 PMC2763872

[115] Thomas JW Hulbert C. Somatically mutated B cell pool provides precursors for insulin antibodies. J Immunol (1996) 157(2):763–71. 8752927

[116] Pauken KE Linehan JL Spanier JA Sahli NL Kalekar LA Binstadt BA Cutting edge: type 1 diabetes occurs despite robust anergy among endogenous insulin-specific CD4 T cells in NOD mice. J Immunol (2013) 191(10):4913–7. 10.4049/jimmunol.1301927 24123682 PMC3987747

[117] Martinez RJ Zhang N Thomas SR Nandiwada SL Jenkins MK Binstadt BA Arthritogenic self-reactive CD4+ T cells acquire an FR4hiCD73hi anergic state in the presence of Foxp3+ regulatory T cells. J Immunol (2012) 188(1):170–81. 10.4049/jimmunol.1101311 22124124 PMC3244540

[118] Kitagawa Y Sakaguchi S. Molecular control of regulatory T cell development and function. Curr Opin Immunol (2017) 49:64–70. 10.1016/j.coi.2017.10.002 29065384

[119] Johnson MC Garland AL Nicolson SC Li C Samulski RJ Wang B Beta-cell-specific IL-2 therapy increases islet Foxp3+Treg and suppresses type 1 diabetes in NOD mice. Diabetes (2013) 62(11):3775–84. 10.2337/db13-0669 23884888 PMC3806588

[120] Davies JD Leong LY Mellor A Cobbold SP Waldmann H. T cell suppression in transplantation tolerance through linked recognition. J Immunol (1996) 156(10):3602–7. 8621893

[121] Waldmann H Cobbold S. How do monoclonal antibodies induce tolerance? A role for infectious tolerance? Annu Rev Immunol (1998) 16:619–44. 10.1146/annurev.immunol.16.1.619 9597144

[122] Anderton SM Wraith DC. Hierarchy in the ability of T cell epitopes to induce peripheral tolerance to antigens from myelin. Eur J Immunol (1998) 28(4):1251–61. 10.1002/(SICI)1521-4141(199804)28:04<1251::AID-IMMU1251>3.0.CO;2-O 9565365

[123] Ito T Yamada A Batal I Yeung MY McGrath MM Sayegh MH The limits of linked suppression for regulatory T cells. Front Immunol (2016) 7:82. 10.3389/fimmu.2016.00082 27014262 PMC4783400

[124] Itoh A Ridgway WM. Targeting innate immunity to downmodulate adaptive immunity and reverse type 1 diabetes. Immunotargets Ther (2017) 6:31–8. 10.2147/ITT.S117264 28580341 PMC5448691

[125] Reed JC Herold KC. Thinking bedside at the bench: the NOD mouse model of T1DM. Nat Rev Endocrinol (2015) 11(5):308–14. 10.1038/nrendo.2014.236 25623120 PMC4523382

[126] Prasad S Xu D Miller SD. Tolerance strategies employing antigen-coupled apoptotic cells and carboxylated PLG nanoparticles for the treatment of type 1 diabetes. Rev Diabet Stud (2012) 9(4):319–27. 10.1900/RDS.2012.9.319 23804269 PMC3740699

[127] Mahne AE Klementowicz JE Chou A Nguyen V Tang Q. Therapeutic regulatory T cells subvert effector T cell function in inflamed islets to halt autoimmune diabetes. J Immunol (2015) 194(7):3147–55. 10.4049/jimmunol.1402739 25732730 PMC4369402

[128] Tang Q Adams JY Penaranda C Melli K Piaggio E Sgouroudis E Central role of defective interleukin-2 production in the triggering of islet autoimmune destruction. Immunity (2008) 28(5):687–97. 10.1016/j.immuni.2008.03.016 18468463 PMC2394854

[129] Grinberg-Bleyer Y Baeyens A You S Elhage R Fourcade G Gregoire S IL-2 reverses established type 1 diabetes in NOD mice by a local effect on pancreatic regulatory T cells. J Exp Med (2010) 207(9):1871–8. 10.1084/jem.20100209 20679400 PMC2931175

[130] Marek-Trzonkowska N Mysliwiec M Dobyszuk A Grabowska M Techmanska I Juscinska J Administration of CD4+CD25highCD127- regulatory T cells preserves beta-cell function in type 1 diabetes in children. Diabetes Care (2012) 35(9):1817–20. 10.2337/dc12-0038 22723342 PMC3425004

[131] Marek-Trzonkowska N Myśliwiec M Dobyszuk A Grabowska M Derkowska I Juścińska J Therapy of type 1 diabetes with CD4(+)CD25(high)CD127-regulatory T cells prolongs survival of pancreatic islets – results of one year follow-up. Clin Immunol (2014) 153(1):23–30. 10.1016/j.clim.2014.03.016 24704576

[132] Putnam AL Brusko TM Lee MR Liu W Szot GL Ghosh T Expansion of human regulatory T-cells from patients with type 1 diabetes. Diabetes (2009) 58(3):652–62. 10.2337/db08-1168 19074986 PMC2646064

[133] Bluestone JA Buckner JH Fitch M Gitelman SE Gupta S Hellerstein MK Type 1 diabetes immunotherapy using polyclonal regulatory T cells. Sci Transl Med (2015) 7(315):315ra189. 10.1126/scitranslmed.aad4134 26606968 PMC4729454

[134] Seay HR Putnam AL Cserny J Posgai AL Rosenau EH Wingard JR Expansion of human Tregs from cryopreserved umbilical cord blood for GMP-compliant autologous adoptive cell transfer therapy. Mol Ther Methods Clin Dev (2017) 4:178–91. 10.1016/j.omtm.2016.12.003 28345003 PMC5363324

[135] Megaw R Dhillon B. Stem cell therapies in the management of diabetic retinopathy. Curr Diab Rep (2014) 14(7):498. 10.1007/s11892-014-0498-9 24796933

[136] Mora-Fernández C Domínguez-Pimentel V de Fuentes MM Górriz JL Martínez-Castelao A Navarro-González JF Diabetic kidney disease: from physiology to therapeutics. J Physiol (2014) 592(18):3997–4012. 10.1113/jphysiol.2014.272328 24907306 PMC4198010

[137] Aslam A Singh J Rajbhandari S. Pathogenesis of painful diabetic neuropathy. Pain Res Treat (2014) 2014:412041. 10.1155/2014/412041 24891949 PMC4026988

[138] Letonja M Petrovic D. Is diabetic cardiomyopathy a specific entity? World J Cardiol (2014) 6(1):8–13. 10.4330/wjc.v6.i1.8 24527183 PMC3920165

[139] Boudina S Abel ED. Diabetic cardiomyopathy, causes and effects. Rev Endocr Metab Disord (2010) 11(1):31–9. 10.1007/s11154-010-9131-7 20180026 PMC2914514

[140] Mukherjee G Chaparro RJ Schloss J Smith C Bando CD DiLorenzo TP. Glucagon-reactive islet-infiltrating CD8 T cells in NOD mice. Immunology (2015) 144(4):631–40. 10.1111/imm.12415 25333865 PMC4368169

[141] Mundinger TO Mei Q Foulis AK Fligner CL Hull RL Taborsky GJ Jr. Human type 1 diabetes is characterized by an early, marked, sustained, and islet-selective loss of sympathetic nerves. Diabetes (2016) 65(8):2322–30. 10.2337/db16-0284 27207540 PMC4955989

[142] McLaughlin KA Richardson CC Ravishankar A Brigatti C Liberati D Lampasona V Identification of tetraspanin-7 as a target of autoantibodies in type 1 diabetes. Diabetes (2016) 65(6):1690–8. 10.2337/db15-1058 26953162

[143] Pagliuca FW Millman JR Gürtler M Segel M Van Dervort A Ryu JH Generation of functional human pancreatic beta cells in vitro. Cell (2014) 159(2):428–39. 10.1016/j.cell.2014.09.040 25303535 PMC4617632

[144] Rezania A Bruin JE Arora P Rubin A Batushansky I Asadi A Reversal of diabetes with insulin-producing cells derived in vitro from human pluripotent stem cells. Nat Biotechnol (2014) 32(11):1121–33. 10.1038/nbt.3033 25211370

[145] Bose B Sudheer PS In vitro differentiation of pluripotent stem cells into functional beta islets under 2D and 3D culture conditions and in vivo preclinical validation of 3D islets. Methods Mol Biol (2016) 1341:257–84. 10.1007/7651_2015_230 25783769

[146] Rajaei B Shamsara M Amirabad LM Massumi M Sanati MH. Pancreatic endoderm-derived from diabetic patient-specific induced pluripotent stem cell generates glucose-responsive insulin-secreting cells. J Cell Physiol (2017) 232(10):2616–25. 10.1002/jcp.25459 27306424

[147] Ryan EA Lakey JR Rajotte RV Korbutt GS Kin T Imes S Clinical outcomes and insulin secretion after islet transplantation with the Edmonton protocol. Diabetes (2001) 50(4):710–9. 10.2337/diabetes.50.4.710 11289033

[148] Ryan EA Paty BW Senior PA Bigam D Alfadhli E Kneteman NM Five-year follow-up after clinical islet transplantation. Diabetes (2005) 54(7):2060–9. 10.2337/diabetes.54.7.2060 15983207

[149] Ricordi C Goldstein JS Balamurugan AN Szot GL Kin T Liu C National Institutes of Health-Sponsored Clinical Islet Transplantation Consortium phase 3 trial: manufacture of a complex cellular product at eight processing facilities. Diabetes (2016) 65(11):3418–28. 10.2337/db16-0234 27465220 PMC5079635

[150] Shapiro AM Pokrywczynska M Ricordi C. Clinical pancreatic islet transplantation. Nat Rev Endocrinol (2017) 13(5):268–77. 10.1038/nrendo.2016.178 27834384

[151] Assalino M Genevay M Morel P Demuylder-Mischler S Toso C Berney T. Recurrence of type 1 diabetes after simultaneous pancreas-kidney transplantation in the absence of GAD and IA-2 autoantibodies. Am J Transplant (2012) 12(2):492–5. 10.1111/j.1600-6143.2011.03844.x 22151900

[152] Burke GW III Vendrame F Pileggi A Ciancio G Reijonen H Pugliese A. Recurrence of autoimmunity following pancreas transplantation. Curr Diab Rep (2011) 11(5):413–9. 10.1007/s11892-011-0206-y 21660419 PMC4018301

[153] Chakravarthy H Gu X Enge M Dai X Wang Y Damond N Converting adult pancreatic islet alpha cells into beta cells by targeting both Dnmt1 and Arx. Cell Metab (2017) 25(3):622–34. 10.1016/j.cmet.2017.01.009 28215845 PMC5358097

[154] Burke GW III Vendrame F Virdi SK Ciancio G Chen L Ruiz P Lessons from pancreas transplantation in type 1 diabetes: recurrence of islet autoimmunity. Curr Diab Rep (2015) 15(12):121. 10.1007/s11892-015-0691-5 26547222

[155] Chang CA Lawrence MC Naziruddin B. Current issues in allogeneic islet transplantation. Curr Opin Organ Transplant (2017) 22(5):437–43. 10.1097/MOT.0000000000000448 28692442

[156] Reffet S Thivolet C. Immunology of pancreatic islet transplantation. Diabetes Metab (2006) 32(5 Pt 2):523–6. 10.1016/S1262-3636(06)72805-1 17130811

[157] Lin CM Gill RG. Direct and indirect allograft recognition: pathways dictating graft rejection mechanisms. Curr Opin Organ Transplant (2016) 21(1):40–4. 10.1097/MOT.0000000000000263 26575853 PMC4701596

[158] Sherman LA Chattopadhyay S. The molecular basis of allorecognition. Annu Rev Immunol (1993) 11:385–402. 10.1146/annurev.iy.11.040193.002125 8476567

[159] Brennan TV Jaigirdar A Hoang V Hayden T Liu FC Zaid H Preferential priming of alloreactive T cells with indirect reactivity. Am J Transplant (2009) 9(4):709–18. 10.1111/j.1600-6143.2009.02578.x 19344462 PMC5990255

[160] Frank R Molina MR Goldberg LR Wald JW Kamoun M Lal P. Circulating donor-specific anti-human leukocyte antigen antibodies and complement C4d deposition are associated with the development of cardiac allograft vasculopathy. Am J Clin Pathol (2014) 142(6):809–15. 10.1309/AJCPTLBEU5BQ8SHN 25389335

[161] Zhao Y Scott NA Quah HS Krishnamurthy B Bond F Loudovaris T Mouse pancreatic beta cells express MHC class II and stimulate CD4(+) T cells to proliferate. Eur J Immunol (2015) 45(9):2494–503. 10.1002/eji.201445378 25959978

[162] Makhlouf L Kishimoto K Smith RN Abdi R Koulmanda M Winn HJ The role of autoimmunity in islet allograft destruction: major histocompatibility complex class II matching is necessary for autoimmune destruction of allogeneic islet transplants after T-cell costimulatory blockade. Diabetes (2002) 51(11):3202–10. 10.2337/diabetes.51.11.3202 12401711

[163] Kupfer TM Crawford ML Pham K Gill RG. MHC-mismatched islet allografts are vulnerable to autoimmune recognition in vivo. J Immunol (2005) 175(4):2309–16. 10.4049/jimmunol.175.4.2309 16081800

[164] Makhlouf L Yamada A Ito T Abdi R Ansari MJ Khuong CQ Allorecognition and effector pathways of islet allograft rejection in normal versus nonobese diabetic mice. J Am Soc Nephrol (2003) 14(8):2168–75. 10.1097/01.ASN.0000079041.15707.A9 12874472

[165] Nicolls MR Coulombe M Beilke J Gelhaus HC Gill RG. CD4-dependent generation of dominant transplantation tolerance induced by simultaneous perturbation of CD154 and LFA-1 pathways. J Immunol (2002) 169(9):4831–9. 10.4049/jimmunol.169.9.4831 12391193

[166] Gerling IC Friedman H Greiner DL Shultz LD Leiter EH. Multiple low-dose streptozocin-induced diabetes in NOD-scid/scid mice in the absence of functional lymphocytes. Diabetes (1994) 43(3):433–40. 10.2337/diab.43.3.433 8314017

[167] Lukic ML Stosic-Grujicic S Shahin A. Effector mechanisms in low-dose streptozotocin-induced diabetes. Dev Immunol (1998) 6(1–2):119–28. 10.1155/1998/92198 9716913 PMC2276003

[168] Gala-Lopez B Pepper AR Shapiro AM. Biologic agents in islet transplantation. Curr Diab Rep (2013) 13(5):713–22. 10.1007/s11892-013-0414-8 23918618

[169] Kimelman M Brandacher G. Trends in immunosuppression after pancreas transplantation: what is in the pipeline? Curr Opin Organ Transplant (2013) 18(1):76–82. 10.1097/MOT.0b013e32835c6eda 23254700

[170] Wang S Zhang X Zhang L Bryant J Kheradmand T Hering BJ Preemptive tolerogenic delivery of donor antigens for permanent allogeneic islet graft protection. Cell Transplant (2015) 24(6):1155–65. 10.3727/096368914X681027 24759564 PMC4206673

[171] Nanji SA Hancock WW Luo B Schur CD Pawlick RL Zhu LF Costimulation blockade of both inducible costimulator and CD40 ligand induces dominant tolerance to islet allografts and prevents spontaneous autoimmune diabetes in the NOD mouse. Diabetes (2006) 55(1):27–33. 10.2337/diabetes.55.01.06.db04-1154 16380473

[172] Ferrer IR Wagener ME Song M Kirk AD Larsen CP Ford ML. Antigen-specific induced Foxp3+ regulatory T cells are generated following CD40/CD154 blockade. Proc Natl Acad Sci U S A (2011) 108(51):20701–6. 10.1073/pnas.1105500108 22143783 PMC3251074

[173] Beilke JN Kuhl NR Van Kaer L Gill RG. NK cells promote islet allograft tolerance via a perforin-dependent mechanism. Nat Med (2005) 11(10):1059–65. 10.1038/nm1296 16155578

[174] Nicolls MR Coulombe M Gill RG. The basis of immunogenicity of endocrine allografts. Crit Rev Immunol (2001) 21(1–3):87–101. 10.1615/CritRevImmunol.v21.i1-3.60 11642616

[175] Coulombe M Yang H Wolf LA Gill RG. Tolerance to antigen-presenting cell-depleted islet allografts is CD4 T cell dependent. J Immunol (1999) 162(5):2503–10. 10072489

[176] Yolcu ES Zhao H Bandura-Morgan L Lacelle C Woodward KB Askenasy N Pancreatic islets engineered with SA-FasL protein establish robust localized tolerance by inducing regulatory T cells in mice. J Immunol (2011) 187(11):5901–9. 10.4049/jimmunol.1003266 22068235 PMC3232043

[177] El Khatib MM Sakuma T Tonne JM Mohamed MS Holditch SJ Lu B Beta-cell-targeted blockage of PD1 and CTLA4 pathways prevents development of autoimmune diabetes and acute allogeneic islets rejection. Gene Ther (2015) 22(5):430–8. 10.1038/gt.2015.18 25786871 PMC4520544

[178] Moore DJ Noorchashm H Lin TH Greeley SA Naji A. NOD B-cells are insufficient to incite T-cell-mediated anti-islet autoimmunity. Diabetes (2005) 54(7):2019–25. 10.2337/diabetes.54.7.2019 15983202

[179] Zheng XX Sánchez-Fueyo A Sho M Domenig C Sayegh MH Strom TB. Favorably tipping the balance between cytopathic and regulatory T cells to create transplantation tolerance. Immunity (2003) 19(4):503–14. 10.1016/S1074-7613(03)00259-0 14563315

[180] Markees TG Serreze DV Phillips NE Sorli CH Gordon EJ Shultz LD NOD mice have a generalized defect in their response to transplantation tolerance induction. Diabetes (1999) 48(5):967–74. 10.2337/diabetes.48.5.967 10331399

[181] Li L Wang B Frelinger JA Tisch R. T-cell promiscuity in autoimmune diabetes. Diabetes (2008) 57(8):2099–106. 10.2337/db08-0383 18492786 PMC2494676

[182] Molano RD Berney T Li H Cattan P Pileggi A Vizzardelli C Prolonged islet graft survival in NOD mice by blockade of the CD40-CD154 pathway of T-cell costimulation. Diabetes (2001) 50(2):270–6. 10.2337/diabetes.50.2.270 11272136

[183] Wang Y Pontesilli O Gill RG La Rosa FG Lafferty KJ. The role of CD4+ and CD8+ T cells in the destruction of islet grafts by spontaneously diabetic mice. Proc Natl Acad Sci U S A (1991) 88(2):527–31. 10.1073/pnas.88.2.527 1899142 PMC50844

[184] Drage M Zaccone P Phillips JM Nicoletti F Dawson J Andrew Bradley J Nondepleting anti-CD4 and soluble interleukin-1 receptor prevent autoimmune destruction of syngeneic islet grafts in diabetic NOD mice. Transplantation (2002) 74(5):611–9. 10.1097/00007890-200209150-00005 12352875

[185] Berney T Pileggi A Molano RD Poggioli R Zahr E Ricordi C The effect of simultaneous CD154 and LFA-1 blockade on the survival of allogeneic islet grafts in nonobese diabetic mice. Transplantation (2003) 76(12):1669–74. 10.1097/01.TP.0000092525.17025.D0 14688513

[186] Naji A Silvers WK Bellgrau D Barker CF. Spontaneous diabetes in rats: destruction of islets is prevented by immunological tolerance. Science (1981) 213(4514):1390–2. 10.1126/science.6791286 6791286

[187] Naji A Bellgrau D Anderson A Silvers WK Barker CF. Transplantation of islets and bone marrow cells to animals with immune insulitis. Diabetes (1982) 31(Suppl 4):84–91. 10.2337/diab.31.4.S84 6819968

[188] Naji A Silvers WK Kimura H Bellgrau D Markmann JF Barker CF. Analytical and functional studies on the T cells of untreated and immunologically tolerant diabetes-prone BB rats. J Immunol (1983) 130(5):2168–72. 6220083

[189] Prowse SJ Bellgrau D Lafferty KJ. Islet allografts are destroyed by disease occurrence in the spontaneously diabetic BB rat. Diabetes (1986) 35(1):110–4. 10.2337/diab.35.1.110 3079713

[190] Langsford D Dwyer K. Dysglycemia after renal transplantation: definition, pathogenesis, outcomes and implications for management. World J Diabetes (2015) 6(10):1132–51. 10.4239/wjd.v6.i10.1132 26322159 PMC4549664

[191] Oling V Marttila J Ilonen J Kwok WW Nepom G Knip M GAD65- and proinsulin-specific CD4+ T-cells detected by MHC class II tetramers in peripheral blood of type 1 diabetes patients and at-risk subjects. J Autoimmun (2005) 25(3):235–43. 10.1016/j.jaut.2005.09.018 16263242

[192] Yang J Danke N Roti M Huston L Greenbaum C Pihoker C CD4+ T cells from type 1 diabetic and healthy subjects exhibit different thresholds of activation to a naturally processed proinsulin epitope. J Autoimmun (2008) 31(1):30–41. 10.1016/j.jaut.2008.02.001 18385016

[193] Yang J Chow IT Sosinowski T Torres-Chinn N Greenbaum CJ James EA Autoreactive T cells specific for insulin B:11-23 recognize a low-affinity peptide register in human subjects with autoimmune diabetes. Proc Natl Acad Sci U S A (2014) 111(41):14840–5. 10.1073/pnas.1416864111 25267644 PMC4205657

[194] Spanier JA Sahli NL Wilson JC Martinov T Dileepan T Burrack AL Increased effector memory insulin-specific CD4+T-cells correlate with insulin autoantibodies in recent-onset type 1 diabetic patients. Diabetes (2017) 66(12):3051–60. 10.2337/db17-0666 28842400 PMC5697953

[195] Antkowiak PF Tersey SA Carter JD Vandsburger MH Nadler JL Epstein FH Noninvasive assessment of pancreatic beta-cell function in vivo with manganese-enhanced magnetic resonance imaging. Am J Physiol Endocrinol Metab (2009) 296(3):E573–8. 10.1152/ajpendo.90336.2008 19116376 PMC2660140

[196] Gaglia JL Guimaraes AR Harisinghani M Turvey SE Jackson R Benoist C Noninvasive imaging of pancreatic islet inflammation in type 1A diabetes patients. J Clin Invest (2011) 121(1):442–5. 10.1172/JCI44339 21123946 PMC3007157

[197] Demeester S Keymeulen B Kaufman L Van Dalem A Balti EV Van de Velde U Preexisting insulin autoantibodies predict efficacy of otelixizumab in preserving residual beta-cell function in recent-onset type 1 diabetes. Diabetes Care (2015) 38(4):644–51. 10.2337/dc14-1575 25583753 PMC4370324

[198] Akirav EM Lebastchi J Galvan EM Henegariu O Akirav M Ablamunits V Detection of beta cell death in diabetes using differentially methylated circulating DNA. Proc Natl Acad Sci U S A (2011) 108(47):19018–23. 10.1073/pnas.1111008108 22074781 PMC3223447

[199] Olsen JA Kenna LA Spelios MG Hessner MJ Akirav EM Circulating differentially methylated amylin DNA as a biomarker of beta-cell loss in type 1 diabetes. PLoS One (2016) 11(4):e0152662 10.1371/journal.pone.0152662 27111653 PMC4844136

[200] Su CA Iida S Abe T Fairchild RL. Endogenous memory CD8 T cells directly mediate cardiac allograft rejection. Am J Transplant (2014) 14(3):568–79. 10.1111/ajt.12605 24502272 PMC3947453

[201] Gorbacheva V Fan R Fairchild RL Baldwin WM III Valujskikh A. Memory CD4 T cells induce antibody-mediated rejection of renal allografts. J Am Soc Nephrol (2016) 27(11):3299–307. 10.1681/ASN.2015080848 27020853 PMC5084884

